# Supplementary material for: Tensorial stress-plastic strain fields in α - ω Zr mixture, transformation kinetics, and friction in diamond-anvil cell
Source: Nat Commun. 2023 Sep 23;14:5955. doi: 10.1038/s41467-023-41680-1 (PMC10517986; doi:10.1038/s41467-023-41680-1)
Supplement: Supplementary file 1 — Supplementary Information [file 41467_2023_41680_MOESM1_ESM.pdf]

Supplementary Information

Tensorial stress-plastic strain fields in α - ω Zr mixture, transformation kinetics, and friction in diamond anvil cell

Valery I. Levitas[1,2,3*], Achyut Dhar[*1], K.K. Pandey[4]

[1] Department of Aerospace Engineering, Iowa State University, Ames, IA 50011, USA
[2] Department of Mechanical Engineering, Iowa State University, Ames, IA 50011, USA
[3] Ames National Laboratory, Division of Materials Science and Engineering, Ames, IA 50011, USA
[4] High Pressure and Synchrotron Radiation Physics Division, Bhabha Atomic Research Centre, Bombay, Mumbai-400085, India

This PDF file includes:

1. Coupled experimental-analytical-computational approaches for finding stress and plastic strain tensor fields and friction rules in a sample compressed in DAC
2. Coupled Experimental-Analytical (CEA) approach
3. The reasons for the difference between stresses and elastic strains from Rietveld refinement and CEA approach
4. FEM simulations
5. Friction stress and rules for α- and ω-Zr and their mixture
6. Pressure-dependence of the yield strengths of $\alpha-$ and $\omega-$Zr
7. Equation of state under hydrostatic loading
Supplementary Figures
Supplementary References

Supplementary Notes

1 Coupled experimental-analytical-computational approaches for finding stress and plastic strain tensor fields and friction rules in a sample compressed in DAC

The flowchart of the interaction between different methods is presented in Supplementary Fig. 1.

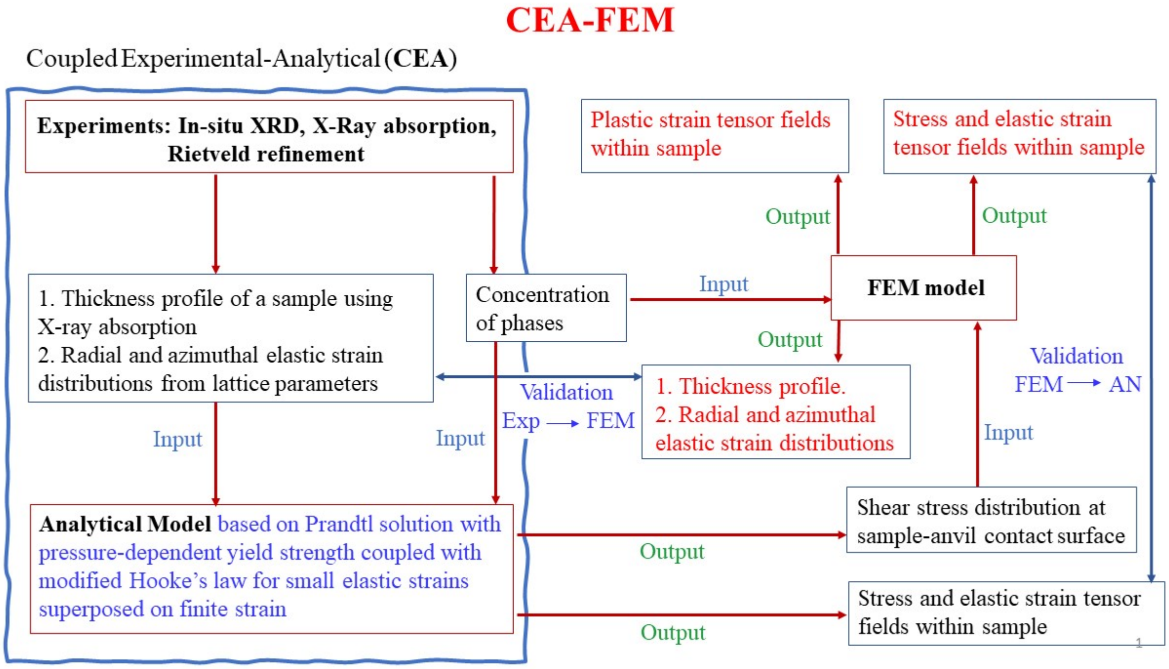

Supplementary Fig. 1: **The flowchart of the interaction between experimental, analytical, and FE methods.**

Experimental methods based on in-situ X-ray diffraction and absorption allow us to find the radial $\bar{E}_{0,rr}$ and the azimuthal $\bar{E}_{0,\theta\theta}$ strain distributions in α-Zr and ω-Zr phases, and concentration of ω phase $c(r)$ (all averaged over the sample thickness), as well as sample thickness profile $h(r)$. These distributions are the input data for our analytical model, which allows us to determine the distribution of the contact friction stress at the sample-anvil surface and 2D fields of all components of the stress and elastic strain tensors in each phase and mixture. The friction stress distribution is utilized as the boundary condition in our FEM problem formulation; the evolution of the concentration of ω phase $c(r)$ is introduced homogeneously along the z-coordinate in our FEM problem formulation. FEM solution delivers all components of stress, elastic, and plastic strain tensors and the sample thickness profile. FEM-based radial $\bar{E}_{0,rr}$ and azimuthal $\bar{E}_{0,\theta\theta}$ strain distributions and the sample thickness profile are compared with experiments to validate FEM modeling, and, consequently, the entire procedure and all fields. All components of the stress and elastic strain tensors in the mixture from the analytical solution are compared with the FEM solution to validate the analytical model.

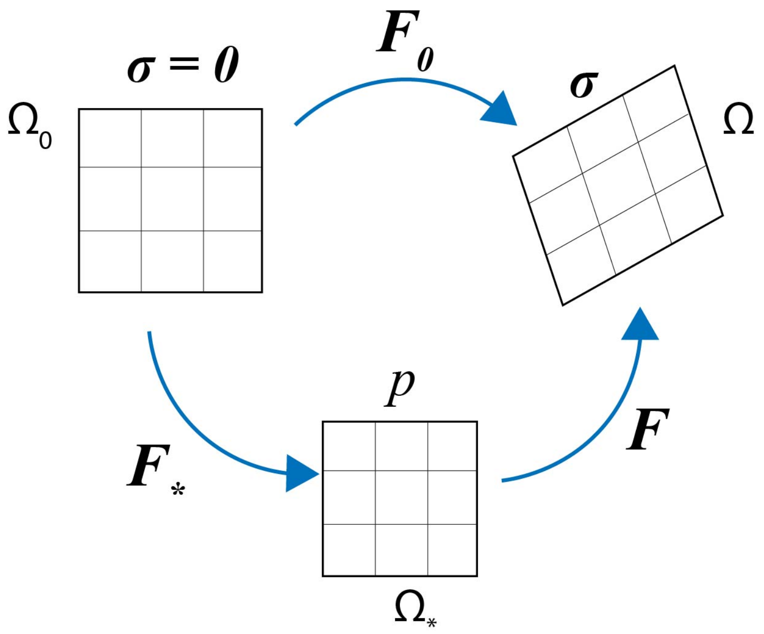

Supplementary Fig. 2: **The stress-free configuration Ω_0, the deformed (current) configuration Ω under the Cauchy stress σ, and an arbitrary intermediate configuration Ω_* with Cauchy stress σ_*. Multiplicative decomposition of the deformation gradient $\boldsymbol{F}_0 = \boldsymbol{F} \cdot \boldsymbol{F}_*$ is valid.**

2 Coupled Experimental-Analytical (CEA) approach

To obtain the analytical solution for stress and elastic strain fields in a sample compressed in DAC, we will make a number of strong and counterintuitive assumptions. Surprisingly, the final analytical solution is in good agreement with the much more precise FEM solution (Fig. 1 and Fig. 2 in the main text), which justifies the admissibility of our assumptions.

2.1 Modified Hooke's law under pressure [2]

Let the total deformation gradient from the undeformed stress-free reference configuration to the current deformed configuration $\boldsymbol{F}_0 = \boldsymbol{F} \cdot \boldsymbol{F}_*$ is decomposed multiplicatively into deformation gradient $\boldsymbol{F}_*$ corresponding to hydrostatically loaded with pressure p intermediate configuration and the deformation gradient $\boldsymbol{F}$ that describes small strains from the intermediate to current configuration (Supplementary Fig. 2). Then the modified Hooke's law is:

$$\boldsymbol{\sigma} = -p(\boldsymbol{F}_*)\boldsymbol{I} + \boldsymbol{B}(p) : \tilde{\boldsymbol{\varepsilon}}; \qquad \tilde{\boldsymbol{\varepsilon}} = (\boldsymbol{F} - \boldsymbol{I})_s; \tag{1}$$

$$\sigma_{ij} = -p(F_{*mn})\delta_{ij} + B_{ijkl}(p)\tilde{\varepsilon}_{lk}; \qquad \tilde{\varepsilon}_{lk} = (F_{lk} - \delta_{lk})_s, \tag{2}$$

where $\boldsymbol{\sigma}$ is the Cauchy (true) stress, pressure $p = -(\sigma_{11} + \sigma_{22} + \sigma_{33})/3$, $\boldsymbol{I}$ is the identity tensor, $\boldsymbol{B}$ is the pressure-dependent elastic moduli tensor that connects Jaumann derivative of the Cauchy stress and the strain-rate (often called the Wallace moduli). Deformation

gradient $\boldsymbol{F}_*(p)$ is measured under hydrostatic DAC experiments and for hexagonal crystals considered here it is:

$$\boldsymbol{F}_*(p) = \begin{bmatrix} F_{*11} & 0 & 0 \\ 0 & F_{*22} & 0 \\ 0 & 0 & F_{*33} \end{bmatrix}, \tag{3}$$

where $F_{*_{11}}(p) = F_{*_{22}}(p) = \frac{a_*}{a_0}$ and $F_{*_{33}}(p) = \frac{c_*}{c_0}$, a_0, c_0 and a_*, c_* are the lattice parameters in the reference and pressurized configurations, respectively. It is important to note that here $\boldsymbol{F}_*$ is written in the local crystal coordinate system, i.e. $F_{*_{11}}$ and $F_{*_{22}}$ are in the basal slip plane, and $F_{*_{33}}$ is normal to the basal slip plane.

The results of our hydrostatic experiments can be approximated as

$$F^{\alpha}_{*11}(p) = 1.00 - 0.00372p + 0.00006p^2; \tag{4}$$

$$F^{\alpha}_{*33}(p) = 1.0 - 0.00333p + 0.00006p^2; \tag{5}$$

$$F^{\omega}_{*11}(p) = 1.00 - 0.00330p + 0.00003p^2, \tag{6}$$

and

$$F^{\omega}_{*33}(p) = 1.0 - 0.00282p + 0.00002p^2. \tag{7}$$

Approximation for $\alpha - Zr$ is valid up to 10 GPa, and for $\omega - Zr$ up to 20 GPa.

Since $\boldsymbol{F}_*$ is diagonal and $\boldsymbol{F}$ describes small strains, then $\boldsymbol{F}_0$ has the form

$$\boldsymbol{F}_0 = \begin{bmatrix} F_{0,11} & \gamma_1 & 0 \\ \gamma_2 & F_{0,22} & 0 \\ 0 & 0 & F_{0,33} \end{bmatrix}, \tag{8}$$

where generally $\gamma_1 \neq \gamma_2$ because of rotations superposed on the shear strains, and we took into account zero shears for the axisymmetric problem.

We need to express $\tilde{\boldsymbol{\varepsilon}}$, which participates in the Hooke's law (1), with $\boldsymbol{F}$ and $\boldsymbol{F}_*$, which are measured. Since $\tilde{\boldsymbol{\varepsilon}}$ is small, therefore,

$$\tilde{\boldsymbol{\varepsilon}} \approx \boldsymbol{E} = \boldsymbol{F}_*^{-1T}(p)\cdot(\boldsymbol{E}_0 - \boldsymbol{E}_*(p))\cdot\boldsymbol{F}_*^{-1}(p); \tag{9}$$

$$\tilde{\varepsilon}_{lk} \approx E_{lk} = F^{-1}_{*ik}(p)(E_{0,ij} - E_{*ij}(p))F^{-1}_{*jl}(p), \tag{10}$$

where $\boldsymbol{E} = 0.5\left(\boldsymbol{F}^T \cdot \boldsymbol{F} - \boldsymbol{I}\right)$ is the Lagrangian strain corresponding to $\boldsymbol{F}$, $\boldsymbol{E}_* = 0.5\left(\boldsymbol{F}_*^T \cdot \boldsymbol{F}_* - \boldsymbol{I}\right)$ and $\boldsymbol{E}_0 = 0.5\left(\boldsymbol{F}_0^T \cdot \boldsymbol{F}_0 - \boldsymbol{I}\right)$ are the Lagrangian strains corresponding to $\boldsymbol{F}_*$ and $\boldsymbol{F}_0$. For

$\boldsymbol{F}_0$ in Eq. (8), we obtain

$$\boldsymbol{E}_0 = 0.5 \begin{bmatrix} F_{0,11}^2 - 1 + \gamma_2^2 & F_{0,11}\gamma_1 + F_{0,22}\gamma_2 & 0 \\ F_{0,11}\gamma_1 + F_{0,22}\gamma_2 & F_{0,22}^2 - 1 + \gamma_1^2 & 0 \\ 0 & 0 & F_{0,33}^2 - 1 \end{bmatrix} \simeq$$

$$0.5 \begin{bmatrix} F_{0,11}^2 - 1 & \gamma & 0 \\ \gamma & F_{0,22}^2 - 1 & 0 \\ 0 & 0 & F_{0,33}^2 - 1 \end{bmatrix}; \qquad \gamma := F_{0,11}\gamma_1 + F_{0,22}\gamma_2, \tag{11}$$

where we neglected small γ_i^2 in comparison with the finite $F_{0,ii}^2 - 1$. Similarly,

$$\boldsymbol{E}_* = 0.5 \begin{bmatrix} F_{*11}^2 - 1 & 0 & 0 \\ 0 & F_{*22}^2 - 1 & 0 \\ 0 & 0 & F_{*33}^2 - 1 \end{bmatrix}. \tag{12}$$

Then, based on Eq. (9),

$$\tilde{\boldsymbol{\varepsilon}} = 0.5 \begin{bmatrix} \frac{E_{0,11} - E_{*11}}{F_{*11}^2} & \frac{\gamma}{F_{*11}F_{*22}} & 0 \\ \frac{\gamma}{F_{*11}F_{*22}} & \frac{E_{0,22} - E_{*22}}{F_{*22}^2} & 0 \\ 0 & 0 & \frac{E_{0,33} - E_{*33}}{F_{*33}^2} \end{bmatrix} = 0.5 \begin{bmatrix} \frac{F_{0,11}^2}{F_{*11}^2} - 1 & \frac{\gamma}{F_{*11}F_{*22}} & 0 \\ \frac{\gamma}{F_{*11}F_{*22}} & \frac{F_{0,22}^2}{F_{*22}^2} - 1 & 0 \\ 0 & 0 & \frac{F_{0,33}^2}{F_{*33}^2} - 1 \end{bmatrix}. \tag{13}$$

With the obtained structure of $\tilde{\boldsymbol{\varepsilon}}$, the Hooke's law for a hydrostatically pre-stressed hexagonal crystals can be split for normal and shear τ stresses:

$$\begin{bmatrix} \sigma_{11} + p \\ \sigma_{22} + p \\ \sigma_{33} + p \end{bmatrix} = \begin{bmatrix} \hat{\sigma}_{11} \\ \hat{\sigma}_{22} \\ \hat{\sigma}_{33} \end{bmatrix} = \begin{bmatrix} B_{11} & B_{12} & B_{13} \\ B_{12} & B_{22} & B_{23} \\ B_{13} & B_{23} & B_{33} \end{bmatrix} \begin{bmatrix} \tilde{\varepsilon}_{11} \\ \tilde{\varepsilon}_{22} \\ \tilde{\varepsilon}_{33} \end{bmatrix}; \qquad \tau = \tau_{13} = 0.5 B_{44} \frac{\gamma}{F_{*11}F_{*22}}, \tag{14}$$

where the simplifications due to hexagonal symmetry of B_{ij} ($B_{11} = B_{22}$, $B_{13} = B_{23}$, $B_{44} = B_{55}$, and $B_{66} = 0.5(B_{11} - B_{12})$) were not yet applied. Since XRD does not measure elastic shear strains, equation for shear components will not be used in further derivations. However, after we will find field of the shear stress τ in the sample, we can use the second Eq. (14) to find elastic shear stain γ.

Pressure-dependence of the single crystal elastic moduli B_{ij} was approximated by a quadratic polynomial with parameters given for α-Zr in Table 1 and for the effective elastic moduli B_{ij}^e for ω-Zr in Table 2. They were produced by combining published experimental and first-principle results ([3] for α-Zr and [4] for ω-Zr) and implementing consistency conditions (see [2]) with our hydrostatic experiments; i.e., elastic moduli B_{ij} reproduce generalized equations of state Eqs. (4)- (7). Effective elastic moduli are defined in Eq. (66) with allowing for an actual orientation of a single crystal with respect to the coordinate system and some additional symmetry requirements.

Note that small strain $\tilde{\boldsymbol{\varepsilon}}$ causes change in pressure. We can iteratively update p (and corresponding $\boldsymbol{F}_*$) in the intermediate configuration, so that it coincides with the pressure in the current configuration. Then strain $\tilde{\boldsymbol{\varepsilon}}$ will produce deviatoric stress, which is limited

Supplementary Table 1: Elastic constants and their pressure derivatives for α-Zr that meet the consistency conditions.

α-Zr	B_{11}	B_{33}	B_{12}	B_{13}	B_{44}
B_{ij} (GPa)	141.3	159.28	70.97	62.94	32.14
dB_{ij}/dp	2.86	3.04	2.44	2.86	-0.22
d^2B_{ij}/dp^2	0.14	0.176	0.12	0.12	0

Supplementary Table 2: Effective elastic constants and their pressure derivatives for ω-Zr that satisfy the consistency conditions.

ω-Zr	B^e_{11}	B^e_{33}	B^e_{13}
B^e_{ij} (GPa)	169.95	168	72.67
dB^e_{ij}/dp	1.84	2.02	1.47
$d^2B^e_{ij}/dp^2$	0.06	0.076	0.068

by the yield strength. Therefore, strain $\tilde{\boldsymbol{\varepsilon}}$ is also limited and is small in comparison with $\boldsymbol{E}_*$, which does not have any constraints.

2.2 Approximate analytical solution of axisymmetric problem on compression of a sample

We consider a polycrystalline material compressed by diamond anvils in axisymmetric formulation. We assume that macroscopically material behaves like perfectly plastic and isotropic. Such behavior can be achieved after large-enough preliminary plastic deformation [5, 6].

System of equations and assumptions. The pressure-dependent von Mises yield condition (i.e., Drucker-Prager yield condition) is assumed

$$(\sigma_{11}-\sigma_{22})^2+(\sigma_{11}-\sigma_{33})^2+(\sigma_{22}-\sigma_{33})^2+6\tau_{13}^2=2\sigma_y^2(p)=6\tau_y^2(p), \qquad (15)$$

where σ_{33}, σ_{11} and σ_{22} are the normal stress components along the load (vertical), radial and azimuthal directions, respectively (Supplementary Fig. 3), $\tau=\tau_{13}$ is the shear stress, and σ_y and τ_y are the yield strengths in compression and shear, respectively. Based on experimental results for α- and ω-Zr,

$$\sigma_y=\sqrt{3}\tau_y=\sigma_y^0+bp, \qquad (16)$$

where constants σ_y^0 and b are from Supplementary Fig. 9. Equilibrium equations are

$$\frac{\partial\sigma_{11}}{\partial r}+\frac{\partial\tau_{13}}{\partial z}+\frac{\sigma_{11}-\sigma_{22}}{r}=0 \quad \text{in radial direction;} \qquad (17)$$

$$\frac{\partial\sigma_{33}}{\partial z}+\frac{\partial\tau_{13}}{\partial r}+\frac{\tau_{13}}{r}=0 \qquad \text{in axial direction.} \qquad (18)$$

The following assumptions are accepted:

1. As it approximately follows from DAC experiments and FEM simulations for polycrystals (based on the phenomenological flow theory of plasticity)

$$\sigma_{11} = \sigma_{22}. \tag{19}$$

Then the yield condition (15) simplifies to

$$(\sigma_{11} - \sigma_{33})^2 + 3\tau_{13}^2 = \sigma_y^2(p) = 3\tau_y^2(p). \tag{20}$$

2. Stress σ_{33} is independent of z. This does not mean that

$$\frac{\partial \tau_{13}}{\partial r} + \frac{\tau_{13}}{r} = 0 \qquad \rightarrow \qquad \tau_{13} = \tau_0(z)\frac{r_0}{r}, \tag{21}$$

r_0 and $\tau_0(z)$ being arbitrary constants, because for material with pressure-independent yield strength, $\tau_0(z)$ at the contact surface $z = h$ ($h = h(r)$ is the half of the sample thickness profile determined from the experiments) may be equal to the constant τ_y for all r at the contact surface. Approximate independence of σ_{33} of z means that two other terms in Eq. (18) make small contribution to σ_{33}.

Solution. A slightly modified Prandtl's solution (which was for a plane strain problem [7]) for stresses that satisfies equilibrium equations, plasticity condition, and the above assumptions is:

$$\frac{\sigma_{33}}{\tau_y} = \frac{\sigma_{33}^s}{\tau_y} + \frac{mr}{h}; \tag{22}$$

$$\frac{\tau_{13}}{\tau_y} = \frac{mz}{h}; \tag{23}$$

$$\frac{\sigma_{11}}{\tau_y} = \frac{\sigma_{33}^s}{\tau_y} + \frac{mr}{h} + \sqrt{3}\sqrt{1 - \left(\frac{mz}{h}\right)^2} = \frac{\sigma_{33}}{\tau_y} + \sqrt{3}\sqrt{1 - \left(\frac{mz}{h}\right)^2}; \tag{24}$$

$$p = -(2\sigma_{11} + \sigma_{33})/3, \tag{25}$$

where σ_{33}^s is the stress σ_{33} at the symmetry axis $r = 0$ and parameter $0 \leq m(r) \leq 1$ is defined by the value of shear stress τ_c at the contact surface, $\tau_c = m\tau_y$; $m(0) = 0$ at the symmetry axis. The difference with the Prandtl's solution is in multiplier $\sqrt{3}$ instead of 2 in Eq. (24) for σ_{11}. The reason is that we use von Mises condition and $\sigma_{11} = \sigma_{22}$, which results in Eq. (20), while in the Prandtl's solution the Tresca condition along with plane strain assumption lead to the yield condition $(\sigma_{11} - \sigma_{33})^2 + 4\tau_{13}^2 = \sigma_y^2 = 4\tau_y^2$.

Averaging over the sample thickness. For averaging all stresses over the sample thickness, to get transparent analytical results, we have to assume that the yield strength τ_y depends

on the pressure averaged over the sample thickness,

$$\overline{p} = \frac{1}{h}\int_0^h p\,dz, \tag{26}$$

i.e.,

$$\sigma_y = \sqrt{3}\tau_y = \sigma_y^0 + b\overline{p}. \tag{27}$$

Then the stress $\bar{\sigma}_{11}$ averaged over the sample thickness is

$$\overline{\sigma}_{11} = \frac{1}{h}\int_0^h \sigma_{11} dz = \sigma_{33} + (\sigma_y^0 + b\overline{p})\frac{m\sqrt{1-m^2} + \arcsin(m)}{2m}. \tag{28}$$

Averaged pressure

$$\overline{p} = -\frac{2}{3}\overline{\sigma}_{11} - \frac{1}{3}\sigma_{33} = -\sigma_{33} - \frac{2}{3}(\sigma_y^0 + b\overline{p})\frac{m\sqrt{1-m^2} + \arcsin(m)}{2m}, \tag{29}$$

where we substituted Eq. (28) for $\overline{\sigma}_{11}$. Resolving this equation for σ_{33}, we obtain

$$\sigma_{33} = -\overline{p} - \frac{2}{3}(\sigma_y^0 + b\overline{p})\frac{m\sqrt{1-m^2} + \arcsin(m)}{2m}. \tag{30}$$

Substituting σ_{33} from Eq. (30) in Eq. (28) and resolving for $\overline{\sigma}_{11}$, we derive

$$\overline{\sigma}_{11} = -\overline{p} + \frac{1}{3}(\sigma_y^0 + b\overline{p})\frac{m\sqrt{1-m^2} + \arcsin(m)}{2m}. \tag{31}$$

Thus, if friction stress in terms of m and homogeneous along z axis stress σ_{33} are known, $\overline{p}$ and $\overline{\sigma}_{11}$ can be calculated.

Let us analyze the z-dependent part of normal stress and its averaged value:

$$\frac{\sigma_{11} - \sigma_{33}}{\sqrt{3}\tau_y} = \sqrt{1 - \left(\frac{mz}{h}\right)^2}; \qquad \frac{\overline{\sigma}_{11} - \sigma_{33}}{\sqrt{3}\tau_y} = \frac{m\sqrt{1-m^2} + \arcsin(m)}{2m}. \tag{32}$$

Note that the averaged value $\overline{\sigma}_{11} - \overline{\sigma}_{33}$ is much closer to the value of $\sigma_{11} - \sigma_{33}$ at the symmetry plane than at the contact surface. Indeed, at the symmetry plane $(\sigma_{11}(0) - \sigma_{33})/(\sqrt{3}\tau_y) = 1$ for all m; for example, for $m = 1$, we have at the contact surface $(\sigma_{11}(1) - \sigma_{33})/(\sqrt{3}\tau_y) = 0$, while averaged value is $(\overline{\sigma}_{11} - \sigma_{33})/(\sqrt{3}\tau_y) = 0.79$.

Relation between averaged stresses and stresses at the contact surface and symmetry plane. Our next objective is to find relationship between $\overline{\sigma}_{11}$ and $\sigma_{11}(0)$ and $\sigma_{11}(1)$ to be used for interpretation of experimental results. We will use the following identity

$$\overline{\sigma}_{11} = \sigma_{11}(1)w + \sigma_{11}(0)(1-w);$$
$$w(m) = \frac{\overline{\sigma}_{11} - \sigma_{11}(0)}{\sigma_{11}(1) - \sigma_{11}(0)} = \frac{\sqrt{1-m^2} + \arcsin(m)/m - 2}{2(\sqrt{1-m^2} - 1)}, \tag{33}$$

where $w(m)$ is treated as the weight factor, which varies in a narrow range between $w(1) = 1 - \pi/4 \simeq 0.215$ and $w(0) = 1/3$. Since σ_{33} is independent of z, we have similar equation for pressure:

$$\overline{p} = p(1)w + p(0)(1 - w). \tag{34}$$

2.3 Special stress states

Here we considered two main stress states under plastic deformation.

1. Along the symmetry axis ($r = m = 0$) and at the symmetry plane ($z = 0$), shear stress is zero, and the stress components along the load axis (Supplementary Fig. 3) are defined by Eq. (24):

$$\sigma_{11}^s = \sigma_{33}^s + \sigma_y; \qquad \sigma_{11}(0) = \sigma_{22}(0) = \sigma_{33} + \sigma_y, \tag{35}$$

   where we took into account that σ_{33} is assumed to be independent of z. Note that compressive normal stresses are negative. Then for pressure one obtains

$$p^s = p(0) = -(\sigma_{33} + 2/3\sigma_y(\overline{p})). \tag{36}$$

2. At sample-diamond contact surface $z/h = 1$, Eq. (24) results in

$$\sigma_{11}(1) = \sigma_{22}(1) = \sigma_{33} + \sigma_y(\overline{p})\sqrt{1 - m^2}. \tag{37}$$

   and

$$p(1) = -(\sigma_{33} + 2/3\sigma_y(\overline{p})\sqrt{1 - m^2}). \tag{38}$$

   When the friction shear stress reaches the yield strength in shear ($m = 1$), the von Mises yield condition (15) results in

$$\sigma_{11}(1) = \sigma_{22}(1) = \sigma_{33}(1) = -p(1) \tag{39}$$

   without the assumption $\sigma_{11} = \sigma_{22}$.

2.4 Application of the modified Hooke's law for α-Zr with c-axis parallel to the loading axis

The modified Prandtl's solution in Section 2.2 allows us to find stress distributions provided that the boundary conditions $m(r)$ for the contact friction and σ_{33}^s are known, but they are not. We have to find a way to utilize experimentally measured fields $\overline{E}_{0,rr}$ and $\overline{E}_{0,\theta\theta}$ to determine $m(r)$ and finalize our analytical solution. Strong observed texture (with c-axis parallel to the loading axis for α-Zr and to the radial direction for ω-Zr) allows us to utilize single crystal elasticity to find stress distributions in a sample using modified Hooke's law at high pressure. Then the Reuss hypothesis that stresses in all single crystals in the representative

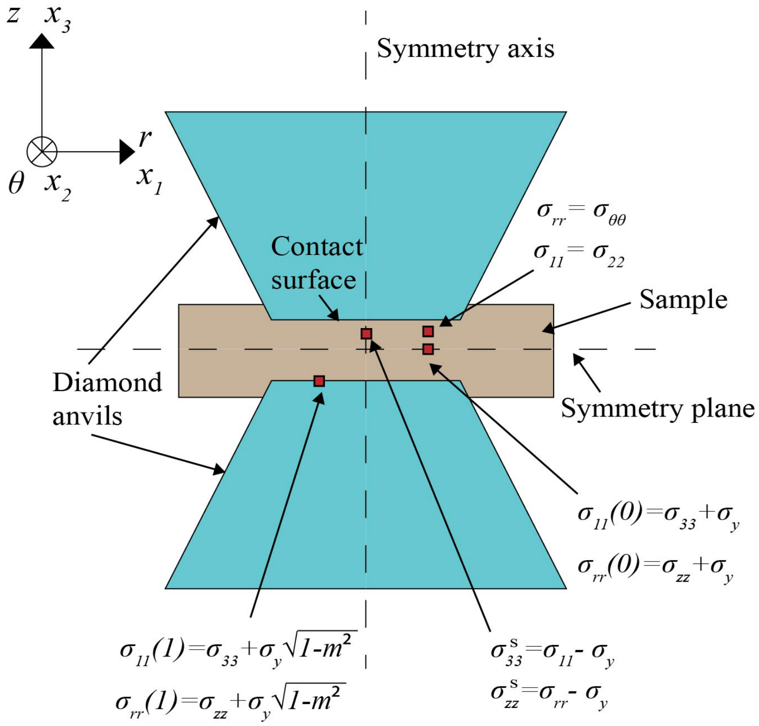

Supplementary Fig. 3: **Schematic illustration of stress states at the symmetry plane, symmetry axis, and the contact surface.**

volume and in polycrystalline aggregate are the same, combined with the simplified mechanical equilibrium condition (59) allow us to connect these stresses and stresses determined by the modified Prandtl's solution and determine $m(r)$, and consequently, all stress and elastic strain fields.

The Hooke's law (14) for hexagonal crystal, in a hydrostatically stressed configuration characterized by pressure p, for normal stresses and strains can be presented in the form

$$\begin{bmatrix}\sigma_{11}+p\\ \sigma_{22}+p\\ \sigma_{33}+p\end{bmatrix}=\begin{bmatrix}\hat{\sigma}_{11}\\ \hat{\sigma}_{22}\\ \hat{\sigma}_{33}\end{bmatrix}=\begin{bmatrix}B_{11} & B_{12} & B_{13}\\ B_{12} & B_{11} & B_{13}\\ B_{13} & B_{13} & B_{33}\end{bmatrix}\begin{bmatrix}\tilde{\varepsilon}_{11}\\ \tilde{\varepsilon}_{22}\\ \tilde{\varepsilon}_{33}\end{bmatrix}. \tag{40}$$

We will not use the second equation (14) for shear strains and stresses because shear strains are not measurable with x-rays and cannot be used for connection to the Prandtl's solution. However, after all stresses, including shear stress will be found, this equation can be used to determine the elastic shear strain.

Using our assumption (19) that $\sigma_{11}=\sigma_{22}$ for a polycrystal and by invoking the Reuss hypothesis, $\sigma_{11}=\sigma_{22}$ in the single crystal is obtained. This equality, using Eq. (40), gives

$$\tilde{\varepsilon}_{11}=\tilde{\varepsilon}_{22} \quad\rightarrow\quad \bar{\tilde{\varepsilon}}_{11}=\bar{\tilde{\varepsilon}}_{22}. \tag{41}$$

From Eq. (13) and $F_{*11}=F_{*22}$ we obtain $F_{0,11}=F_{0,22}$ and $E_{0,11}=E_{0,22}$. Averaging these equations over a sample thickness leads to the experimental observation that $\overline{F}_{0,11}=\overline{F}_{0,22}$ and $\overline{E}_{0,11}=\overline{E}_{0,22}$.

For $\sigma_{11} = \sigma_{22}$, one obtains from Eq. (40)

$$\begin{aligned}\tilde{\varepsilon}_{11}(z/h) &= \tilde{\varepsilon}_{22}(z/h) = k(B_{33}\hat{\sigma}_{11}(z/h) - B_{13}\hat{\sigma}_{33});\\ \tilde{\varepsilon}_{33}(z/h) &= k((B_{11}+B_{12})\hat{\sigma}_{33} - 2B_{13}\hat{\sigma}_{11}(z/h)); \quad k = \left((B_{11}+B_{12})B_{33} - 2B_{13}^2\right)^{-1}.\end{aligned} \tag{42}$$

Averaging over the sample thickness. Averaging Eq. (42) over the thickness, we derive

$$\bar{\tilde{\varepsilon}}_{11} = k(B_{33}\left(\overline{\sigma}_{11} + \overline{p}\right) - B_{13}\left(\overline{\sigma}_{33} + \overline{p}\right)); \tag{43}$$

$$\bar{\tilde{\varepsilon}}_{33} = k((B_{11}+B_{22})\left(\overline{\sigma}_{33} + \overline{p}\right) - 2B_{13}\left(\overline{\sigma}_{11} + \overline{p}\right)). \tag{44}$$

Note that elastic moduli $\boldsymbol{B}$ are considered homogeneous along the thickness direction for simplicity; they are also considered to be functions of averaged through the thickness pressure $\overline{p}$, i.e., $\boldsymbol{B} = \boldsymbol{B}(\overline{p})$, where $\overline{p} = \overline{p}(r)$. To prove for elastic strains relationship similar to Eq. (33) for stresses, let us derive an alternative expression for $\overline{\varepsilon}_{11}$. It follows from Eq. (42)

$$\tilde{\varepsilon}_{11}(0) = k(B_{33}\hat{\sigma}_{11}(0) - B_{13}\hat{\sigma}_{33}(0)) = k(B_{33}(\sigma_{11}(0)+p(0)) - B_{13}(\sigma_{33}+p(0))); \tag{45}$$

$$\tilde{\varepsilon}_{11}(1) = k(B_{33}(\sigma_{11}(1)+p(1)) - B_{13}(\sigma_{33}+p(1))). \tag{46}$$

We remind that the arguments 0 and 1 designate points at the symmetry plane and the contact surface, respectively. Multiplying Eq. (45) by $1-w$ and Eq. (46) by w and adding them, we obtain

$$\begin{aligned}(1-w)\tilde{\varepsilon}_{11}(0) + w\tilde{\varepsilon}_{11}(1) = k[B_{33}\left((1-w)\sigma_{11}(0) + w\sigma_{11}(1)\right) + B_{33}\left((1-w)p(0) + wp(1)\right) - \\ B_{13}\left((1-w)\sigma_{33} + w\sigma_{33} + (1-w)p(0) + wp(1)\right)].\end{aligned} \tag{47}$$

Using Eq. (34) in Eq. (47), we obtain

$$(1-w)\tilde{\varepsilon}_{11}(0) + w\tilde{\varepsilon}_{11}(1) = k[B_{33}(\overline{\sigma}_{11} + \overline{p}) - B_{13}(\sigma_{33} + \overline{p})], \tag{48}$$

where the first equation Eq. (33) was utilized. Comparison of Eq. (48) and Eq. (43) leads to important conclusion that the averaged small imposed strains,

$$\bar{\tilde{\varepsilon}}_{11} = \bar{\tilde{\varepsilon}}_{22} = (1-w)\tilde{\varepsilon}_{11}(0) + w\tilde{\varepsilon}_{11}(1), \tag{49}$$

have similar expressions as the averaged stress $\overline{\sigma}_{11}$ in Eq. (33).This result will be utilized for interpretation of experimental measurements of $\overline{\varepsilon}_{11}$. Similarly, for $\bar{\tilde{\varepsilon}}_{33}$

$$\bar{\tilde{\varepsilon}}_{33} = (1-w)\tilde{\varepsilon}_{33}(0) + w\tilde{\varepsilon}_{33}(1). \tag{50}$$

At the symmetry plane, using $\tilde{\varepsilon}_{11}(0) = \tilde{\varepsilon}_{22}(0)$, $\sigma_{11}(0) = \sigma_{22}(0)$ and Eq. (35) in Eq. (40), we derive

$$\begin{bmatrix}\sigma_{33} + \sigma_y(\overline{p}) + p(0)\\ \sigma_{33} + \sigma_y(\overline{p}) + p(0)\\ \sigma_{33} + p(0)\end{bmatrix} = \begin{bmatrix}B_{11} & B_{12} & B_{13}\\ B_{12} & B_{11} & B_{13}\\ B_{13} & B_{13} & B_{33}\end{bmatrix}\begin{bmatrix}\tilde{\varepsilon}_{11}(0)\\ \tilde{\varepsilon}_{11}(0)\\ \tilde{\varepsilon}_{33}(0)\end{bmatrix}. \tag{51}$$

At the sample-anvil contact surface, using Eqs. (37), we obtain from Eqs. (40)

$$\begin{bmatrix} \sigma_{33} + \sigma_y(\overline{p})\sqrt{1-m^2} + p(1) \\ \sigma_{33} + \sigma_y(\overline{p})\sqrt{1-m^2} + p(1) \\ \sigma_{33} + p(1) \end{bmatrix} = \begin{bmatrix} B_{11} & B_{12} & B_{13} \\ B_{12} & B_{11} & B_{13} \\ B_{13} & B_{13} & B_{33} \end{bmatrix} \begin{bmatrix} \tilde{\varepsilon}_{11}(1) \\ \tilde{\varepsilon}_{11}(1) \\ \tilde{\varepsilon}_{33}(1) \end{bmatrix}. \tag{52}$$

In order to utilize averaged over the thickness elastic strains $\overline{\tilde{\varepsilon}}_{11} = \overline{\tilde{\varepsilon}}_{22}$ (which are related to $\overline{E}_{0,11} = \overline{E}_{0,22}$ and $\overline{F}_{*11} = \overline{F}_{*22}$ measured in experiments through Eq. (10)), let us multiply Eq. (51) by $1-w$, Eq. (52) by w, and combine them:

$$\begin{bmatrix} B_{11} & B_{12} & B_{13} \\ B_{12} & B_{11} & B_{13} \\ B_{13} & B_{13} & B_{33} \end{bmatrix} \begin{bmatrix} \overline{\tilde{\varepsilon}}_{11} \\ \overline{\tilde{\varepsilon}}_{11} \\ \overline{\tilde{\varepsilon}}_{33} \end{bmatrix} = \begin{bmatrix} \sigma_{33} + (1-w)\sigma_y(\overline{p}) + w\sigma_y(\overline{p})\sqrt{1-m^2} + (1-w)p(0) + wp(1) \\ \sigma_{33} + (1-w)\sigma_y(\overline{p})) + w\sigma_y(\overline{p})\sqrt{1-m^2} + (1-w)p(0) + wp(1) \\ \sigma_{33} + (1-w)p(0) + wp(1) \end{bmatrix}$$

$$= \begin{bmatrix} \sigma_{33} + (1-w)\sigma_y(\overline{p}) + w\sigma_y(\overline{p})\sqrt{1-m^2} + \overline{p} \\ \sigma_{33} + (1-w)\sigma_y(\overline{p}) + w\sigma_y(\overline{p})\sqrt{1-m^2} + \overline{p} \\ \sigma_{33} + \overline{p} \end{bmatrix}. \tag{53}$$

In obtaining Eq. (53), Eqs. (34), (49) and (50) were used. The advantage of Eq. (53) is that it includes $\overline{\tilde{\varepsilon}}_{11}$, which will allow us to express all stresses and strains in terms of $\overline{\tilde{\varepsilon}}_{11}$. Inversion of Eqs. (53) gives:

$$\overline{\tilde{\varepsilon}}_{33} = k(B_{11} + B_{12} - 2B_{13})(\sigma_{33} + \overline{p}) - 2kB_{13}\left[(1-w)\sigma_y(\overline{p}) + w\sigma_y(\overline{p})\sqrt{1-m^2}\right]. \tag{54}$$

Also, the third Eq. (53) is

$$\sigma_{33} + \overline{p} = 2B_{13}\overline{\tilde{\varepsilon}}_{11} + B_{33}\overline{\tilde{\varepsilon}}_{33}. \tag{55}$$

Substituting Eq. (55) in Eq. (54) and solving for $\overline{\tilde{\varepsilon}}_{33}$, one finds

$$\overline{\tilde{\varepsilon}}_{33} = (B_{33} - B_{13})^{-1}\left[(B_{11} + B_{12} - 2B_{13})\overline{\tilde{\varepsilon}}_{11} - (1-w)\sigma_y(\overline{p}) - w\sigma_y(\overline{p})\sqrt{1-m^2}\right]. \tag{56}$$

Placing Eq. (56) in Eq. (55), we obtain expression for $\sigma_{33} + \overline{p}$:

$$\sigma_{33} + \overline{p} = \tag{57}$$
$$(B_{13} - B_{33})^{-1}\left[(2B_{13}^2 - B_{11}B_{33} - B_{12}B_{33})\overline{\tilde{\varepsilon}}_{11} + B_{33}\sigma_y(\overline{p})\left(1 - w + w\sqrt{1-m^2}\right)\right].$$

Next, we substitute $\sigma_{33} + \overline{p}$ from Eq. (30) in Eq. (57) and obtain nonlinear equation for $\overline{p}$ (if we assume m to be known):

$$(B_{13} - B_{33})^{-1}\left[(2B_{13}^2 - B_{11}B_{33} - B_{12}B_{33})\overline{\tilde{\varepsilon}}_{11} + B_{33}\sigma_y(\overline{p})\left(1 - w + w\sqrt{1-m^2}\right)\right]$$
$$= -\frac{2}{3}(\sigma_y^0 + b\overline{p})\frac{m\sqrt{1-m^2} + \arcsin(m)}{2m}. \tag{58}$$

Note that B_{ij} are quadratic functions of pressure, and due to nonlinearity of Eq. (58) in B_{ij}, it is strongly nonlinear in $\overline{p}$ and cannot be solved analytically. After numerical solution

of Eq. (58) for $\overline{p}$, Eq. (30) gives numerical value of σ_{33}. Note that since experimentally determined strains $\overline{\varepsilon}_{11} = \overline{\varepsilon}_{22}$ are functions of the radius r, Eq. (57) and solution to Eq. (58) also reproduce the radial dependence of σ_{33} and $\overline{p}$. Then Eqs. (23), (24), and (25) can be used to reproduce z-dependence of the shear stress τ_{13}, normal stresses $\sigma_{11} = \sigma_{22}$, and pressure p. Elastic strains can be found after substituting stresses in Eqs. (42). The next step is to derive the additional equation for m to be combined with Eq. (58).

2.5 Evaluation of friction stress utilizing pressure gradient method

The friction stress τ_c is defined by the simplified mechanical equilibrium equation [5]

$$\frac{d\overline{\sigma}_{11}}{dr} = \frac{\tau_c}{h(r)} = \frac{m(r)\tau_y(\overline{p})}{h(r)}. \tag{59}$$

For solution, we approximated this differential equation with the finite difference equation, using midpoint algorithm, e.g.,

$$\frac{\overline{\sigma}_{11,i+1} - \overline{\sigma}_{11,i-1}}{2\Delta r} = \frac{m_i(\sigma_y^0 + b\overline{p}_i)}{\sqrt{3}h_i}, \tag{60}$$

where subscript i designates value of the function at point r_i. This equation is supplemented with Eqs. (31) and (58) for each radial point r_i:

$$\overline{\sigma}_{11} = -\overline{p} + \frac{1}{3}(\sigma_y^0 + b\overline{p})\frac{m\sqrt{1-m^2} + \arcsin(m)}{2m}. \tag{61}$$

$$\begin{aligned}&(B_{13} - B_{33})^{-1}\left[(2B_{13}^2 - B_{11}B_{33} - B_{12}B_{33})\overline{\overline{\varepsilon}}_{11} + B_{33}\sigma_y(\overline{p})\left(1 - w + w\sqrt{1-m^2}\right)\right]\\ &= -\frac{2}{3}(\sigma_y^0 + b\overline{p})\frac{m\sqrt{1-m^2} + \arcsin(m)}{2m}.\end{aligned} \tag{62}$$

Eqs. (60)-(62) represent system of 3N nonlinear algebraic/trigonometric equations for 3 unknowns m, $\overline{p}$, and $\overline{\sigma}_{11}$ in each of N points along the radial direction r, which we solved numerically. Since for $\overline{\sigma}_{11}$ we have explicit expression, which in practice is substituted in Eq. (60), we have 2N nonlinear algebraic/trigonometric equations for 2 unknowns m and $\overline{p}$ in each of N points. Then Eqs. (23), (24), and (25) can be used to reproduce z-dependence of the shear stress τ_{13}, normal stresses $\sigma_{11} = \sigma_{22}$, and pressure p. Elastic strains can be found after substituting stresses in Eqs. (42).

We can pass to FEM simulations, as one of the boundary conditions, either obtained contact shear stress $\tau_c(r)$ or coefficient $m(r)$. In the latter case, it is more precise to redefine m based on the local pressure $p(1)$, i.e., from $\tau_c(r) = m(r)\tau_y(\overline{p}) = m'(r)\tau_y[p(1)]$ and use in FEM simulations m', because in FEM τ_y at the contact surface depends on $p(1)$. Pressure $p(1)$ can be obtained from Eq. (38), in which σ_{33} is substituted with the expression (30)):

$$p(1) = \overline{p} - \frac{1}{3}\sigma_y(\overline{p})\frac{m\sqrt{1-m^2} - \arcsin(m)}{m}. \tag{63}$$

2.6 Application of the Hooke's law for ω-Zr with c-axis parallel to the radial direction

The modified Hooke's law for hexagonal crystal for normal stresses and strains, when c-axis is parallel to the radial direction 1 can be presented in the form

$$\begin{bmatrix} \sigma_{11}+p \\ \sigma_{22}+p \\ \sigma_{33}+p \end{bmatrix} = \begin{bmatrix} \hat{\sigma}_{11} \\ \hat{\sigma}_{22} \\ \hat{\sigma}_{33} \end{bmatrix} = \begin{bmatrix} B_{33} & B_{13} & B_{13} \\ B_{13} & B_{11} & B_{12} \\ B_{13} & B_{12} & B_{11} \end{bmatrix} \begin{bmatrix} \tilde{\varepsilon}_{11} \\ \tilde{\varepsilon}_{22} \\ \tilde{\varepsilon}_{33} \end{bmatrix}. \tag{64}$$

Our assumption (19) $\sigma_{11} = \sigma_{22}$ for a polycrystal along with the Reuss hypothesis leads to $\sigma_{11} = \sigma_{22}$ for the single crystal. The problem is that this equality substituted in Eq. (64) does not lead to $\tilde{\varepsilon}_{11} = \tilde{\varepsilon}_{22}$ and hence, violates the experimental observation that $\overline{E}_{0,11} = \overline{E}_{0,22}$. That is we need to modify the elastic moduli tensor $\boldsymbol{B}$ for consistency with experiments.

The simplest way to satisfy $\sigma_{11} = \sigma_{22}$ for polycrystal is to assume that for $\tilde{\varepsilon}_{11} = \tilde{\varepsilon}_{22}$ stresses σ^p_{11} and σ^p_{22} for polycrystal are defined as

$$\sigma^p_{11} = \sigma^p_{22} = 0.5(\sigma_{11} + \sigma_{22}) \quad for \quad \tilde{\varepsilon}_{11} = \tilde{\varepsilon}_{22}. \tag{65}$$

This can be imposed by changing the elastic moduli matrix in Eq. (64) with an "effective" elastic modular matrix

$$\begin{bmatrix} \sigma_{11}+p \\ \sigma_{22}+p \\ \sigma_{33}+p \end{bmatrix} = \begin{bmatrix} \hat{\sigma}_{11} \\ \hat{\sigma}_{22} \\ \hat{\sigma}_{33} \end{bmatrix} = \begin{bmatrix} B^e_{11} & B^e_{12} & B^e_{13} \\ B^e_{12} & B^e_{11} & B^e_{13} \\ B^e_{13} & B^e_{13} & B^e_{33} \end{bmatrix} \begin{bmatrix} \tilde{\varepsilon}_{11} \\ \tilde{\varepsilon}_{22} \\ \tilde{\varepsilon}_{33} \end{bmatrix} = \tag{66}$$
$$\begin{bmatrix} 0.5(B_{11}+B_{33}) & B_{13} & 0.5(B_{12}+B_{13}) \\ B_{13} & 0.5(B_{11}+B_{33}) & 0.5(B_{12}+B_{13}) \\ 0.5(B_{12}+B_{13}) & 0.5(B_{12}+B_{13}) & B_{11} \end{bmatrix} \begin{bmatrix} \tilde{\varepsilon}_{11} \\ \tilde{\varepsilon}_{22} \\ \tilde{\varepsilon}_{33} \end{bmatrix}.$$

Thus, we substitute elastic constants in positions 11 and 22 in matrix (64) (i.e., B_{33} and B_{11}) with their average $0.5(B_{11}+B_{33})$, and elastic constants in positions 13 and 23 in matrix (64) (i.e., B_{13} and B_{12}) with their average $0.5(B_{12}+B_{13})$, keeping symmetry of the elasticity matrix. Matrix (66) has 4 independent elastic constants, like matrix (64). However, with such a procedure, we violated equality of elastic moduli in positions 12 and 13 in Eq. (64). To restore this equality (symmetry), we accept

$$\begin{bmatrix} \sigma_{11}+p \\ \sigma_{22}+p \\ \sigma_{33}+p \end{bmatrix} = \begin{bmatrix} B^e_{11} & B^e_{13} & B^e_{13} \\ B^e_{13} & B^e_{11} & B^e_{13} \\ B^e_{13} & B^e_{13} & B^e_{33} \end{bmatrix} \begin{bmatrix} \tilde{\varepsilon}_{11} \\ \tilde{\varepsilon}_{22} \\ \tilde{\varepsilon}_{33} \end{bmatrix} = \tag{67}$$
$$\begin{bmatrix} 0.5(B_{11}+B_{33}) & 0.5(B_{12}+B_{13}) & 0.5(B_{12}+B_{13}) \\ 0.5(B_{12}+B_{13}) & 0.5(B_{11}+B_{33}) & 0.5(B_{12}+B_{13}) \\ 0.5(B_{12}+B_{13}) & 0.5(B_{12}+B_{13}) & B_{11} \end{bmatrix} \begin{bmatrix} \tilde{\varepsilon}_{11} \\ \tilde{\varepsilon}_{22} \\ \tilde{\varepsilon}_{33} \end{bmatrix}.$$

Reduction in number of independent elastic moduli for normal strains from 4 to 3 in transition from a single crystal to textured polycrystal is natural. After such a modification of the $\boldsymbol{B}$-matrix, all equations (42)-(62) for α-Zr can be applied with adding superscript e to

components B_{ij} and imposing $B_{12} = B_{13}$.

2.7 Generalization for the two-phase mixture

Equations for each phase. Radial distribution of volume fraction of the low- and high-pressure phases, averaged over the sample thickness, c_1 and $c_2 = 1 - c_1 = c$, are determined from the experiment using Rietveld refinement. It is independent of the interpretation of stresses and strains and does not participate in the iterations to determine distribution of the stress and elastic strain tensors. Radial distributions of strain $\bar{E}^1_{0,11} = \bar{E}^2_{022}$ is measured in each phase 1 and 2. The modified Hooke's law and plasticity condition are satisfied for each phase separately. We assume

$$m_1 = m_2 = m \tag{68}$$

and that $\sigma^k_{11} = \sigma^k_{22}$, σ^k_{33} is independent of z, and Eqs. (22) - (24) are valid for stresses in each phase. Then Eqs. (22) - (67) are valid for each phase with the elastic constants and the yield strength of each phase, all in terms of experimentally measured elastic strains $\bar{E}^k_{0,11} = \bar{E}^k_{0,22}$ in each phase. That means that for two-phase material, we have to perform the same procedure and use the same equations for each phase separately and find solution in each phase separately. This does not mean that we completely neglect interaction between phases, because the experimentally measured elastic strains $\bar{E}^k_{0,11} = \bar{E}^k_{0,22}$ in each phase do include such an interaction.

Equations for mixture. Since all fields in each phase are known, here we define averaging rules to determine fields in the mixture. We assume for deformation gradient under hydrostatic pressure $\boldsymbol{F}_*$, elastic superposed strains ε_{ii}, and for stresses σ_{ii}

$$\begin{aligned} &\boldsymbol{F}_* = c_1\boldsymbol{F}^1_* + c_2\boldsymbol{F}^2_*; \qquad E_{0,ii} = c_1 E^1_{0,ii} + c_2 E^2_{0,ii}; \qquad \gamma = c_1\gamma^1 + c_2\gamma^2; \\ &\sigma_{ii} = c_1\sigma^1_{ii} + c_2\sigma^2_{ii}; \qquad \tau = c_1\tau^1 + c_2\tau^2; \qquad \tilde{\varepsilon}_{ii} = c_1\tilde{\varepsilon}^1_{ii} + c_2\tilde{\varepsilon}^2_{ii}. \end{aligned} \tag{69}$$

While for stresses the averaging equation is exact, for elastic strain and $\boldsymbol{F}_*$ they represent strong assumption, because $\boldsymbol{F}_*$ and $\tilde{\boldsymbol{\varepsilon}}$ are incompatible separately even in the absence of the plastic and transformation strains, because of heterogeneous pressure distribution, and consequently $\boldsymbol{F}_*$. For shear stress at the contact surface, in particular,

$$\tau_c = m\tau_y = c_1\tau_{c1}(\overline{p}_1) + c_2\tau_{c2}(\overline{p}_2) = m(c_1\tau_{y1}(\overline{p}_1) + c_2\tau_{y2}(\overline{p}_2)), \tag{70}$$

where from we obtain the mixture rule for the pressure-dependent yield strength

$$\begin{aligned} &\tau_y = c_1\tau_{y1}(\overline{p}_1) + c_2\tau_{y2}(\overline{p}_2); \\ &\sigma_y = \sqrt{3}\tau_y = c_1\sigma_{y1}(\overline{p}_1) + c_2\sigma_{y2}(\overline{p}_2) = c_1\sigma^0_{y1} + c_2\sigma^0_{y2} + c_1 b_1\overline{p}_1 + c_2 b_2\overline{p}_2. \end{aligned} \tag{71}$$

2.8 Explicit relationships between $\tilde{\varepsilon}$, $\boldsymbol{E}$ and $\boldsymbol{F}_*$

It follows from Eq. (10) or (13) that

$$\tilde{\varepsilon}_{11} = \tilde{\varepsilon}_{22} = \frac{E_{0,11} - 0.5\left((F_{*11}(p))^2 - 1\right)}{(F_{*11}(p))^2}, \tag{72}$$

where for $\alpha - Zr$ $F_{*11}(p)$ is given by Eq. (4). It is important to note that the $\alpha - Zr$ crystal coordinate system coincides with the global coordinate system and therefore the local $F_{*11}(p)$ coincides with the global in Eq. (72). Averaged over the sample thickness $\bar{\tilde{\varepsilon}}_{11}$, which is present in Eq. (56), can be obtained from Eq. (72) as follows:

$$\bar{\tilde{\varepsilon}}_{11} = \frac{1}{h}\int_0^h \left(\frac{E_{0,11}}{(F_{*11}(p))^2} + \frac{1}{2}\frac{1}{(F_{*11}(p))^2} - \frac{1}{2}\right) dz \tag{73}$$

Based on Eq. (4), we can approximate in the pressure range of $0 - 10$ GPa as $\frac{1}{(F_{*11}(p))^2} = 1 + 0.00668p$. Using this, Eq. (73) can be written as

$$\bar{\tilde{\varepsilon}}_{11} = \frac{0.00668}{h}\int_0^h E_{0,11}p\, dz + \overline{E}_{0,11} + 0.00334\overline{p}. \tag{74}$$

It is impossible to evaluate $\int_0^h E_{0,11}p\, dz$ in terms of $\overline{p}$ and $\overline{E}_{0,11}$ without knowing the distribution of $E_{0,11}$ and therefore, it is approximated as:

$$\frac{1}{h}\int_0^h E_{0,11}p\, dz \simeq \overline{E}_{0,11}\overline{p}. \tag{75}$$

Combination of Eqs. (75) and (74) results in

$$\bar{\tilde{\varepsilon}}_{11} = 0.00668\overline{E}_{0,11}\overline{p} + \overline{E}_{0,11} + 0.00334\overline{p}. \tag{76}$$

As it was already mentioned that the c-axis of ω-Zr crystal is primarily oriented along the global 1 direction and therefore, it is local $F_{*33}(p)$ (rather than the local $F_{*11}(p)$) that coincides with the global 1 direction, unlike for the α-Zr. Also, to enforce $\tilde{\varepsilon}_{11} = \tilde{\varepsilon}_{22}$, given $\overline{E}_{0,11} = \overline{E}_{0,22}$ from experiments, Eq. (72) transforms for ω-Zr as:

$$\tilde{\varepsilon}_{11} = \tilde{\varepsilon}_{22} = \frac{E_{0,11} - 0.5\left(\left(F^{ef}_{*11}(p)\right)^2 - 1\right)}{\left(F^{ef}_{*11}(p)\right)^2}, \tag{77}$$

where, $F^{ef}_{*11} = F^{ef}_{*22} = 0.5(F^{\omega}_{*11} + F^{\omega}_{*33})$. $F^{\omega}_{*11}(p)$ and $F^{\omega}_{*33}(p)$ given in Eqs. (6) and (7) are written here again for convenience:

$$F^{\omega}_{*11}(p) = 1.00 - 0.00330p + 0.00003p^2, \tag{78}$$

and

$$F^{\omega}_{*33}(p) = 1.0 - 0.00282p + 0.00002p^2. \tag{79}$$

Following the same averaging procedure as was done with α-Zr, for a pressure range of $0 - 20$ GPa, the approximation for ω-Zr is

$$\frac{1}{\left(F^{ef}_{*11}(p)\right)^2} = 1.0 + 0.0055p \tag{80}$$

resulting in

$$\bar{\tilde{\varepsilon}}_{11} = \bar{\tilde{\varepsilon}}_{22} = 0.0055\overline{E}_{0,11}\overline{p} + \overline{E}_{0,11} + 0.0028\overline{p}. \tag{81}$$

Likewise, from Eq. (10) for direction 3 we have:

$$E_{0,33} = \tilde{\varepsilon}_{33}\left(F_{*33}(p)\right)^2 + 0.5((F_{*33}(p))^2 - 1). \tag{82}$$

Eq. (82) is not used in further derivations; it just defines the total Lagrangian strain $E_{0,33}$ when $\tilde{\varepsilon}_{33}$ is already found. Since function F_{*33} is given by Eq. (5) for $\alpha - Zr$, it can be approximated by

$$\left(F_{*33}(p)\right)^2 = 1 - 0.00664p + 0.00013p^2. \tag{83}$$

For the rotated $\omega - Zr$ crystal, the $E_{0,33}$ in the global coordinate system is as follows:

$$E_{0,33} = \tilde{\varepsilon}_{33}\left(F^{ef}_{*33}(p)\right)^2 + 0.5\left(\left(F^{ef}_{*33}(p)\right)^2 - 1\right), \tag{84}$$

where $F^{ef}_{*33} = F^{\omega}_{*11}$ and $\left(F^{ef}_{*33}(p)\right)^2 = 1 - 0.0066p + 0.000077p^2$ for the $\omega - Zr$.

For averaging over the thickness, we use p from the Prandtl's solution Eq. (25):

$$p = -\frac{1}{3}\left(2\sigma_{11} + \sigma_{33}\right) = -\sigma_{33} - \frac{2}{3}\sigma_y\sqrt{1 - \left(\frac{mz}{h}\right)^2}. \tag{85}$$

Then

$$p^2 = \sigma_{33}^2 + \frac{4}{9}\sigma_y^2\left(1 - \left(\frac{mz}{h}\right)^2\right) + \frac{4}{3}\sigma_{33}\sigma_y\sqrt{1 - \left(\frac{mz}{h}\right)^2}. \tag{86}$$

After averaging over the thickness we obtain:

$$\overline{p^2} = \sigma_{33}^2 + \frac{4}{9}\sigma_y^2(\overline{p})\left(1 - \frac{m^2}{3}\right) + \frac{4}{3}\sigma_{33}\sigma_y(\overline{p})\frac{m\sqrt{1 - m^2} + sin^{-1}m}{2m}. \tag{87}$$

It is clear that $\overline{p^2} \neq \overline{p}^2$. Using Eq. (82), the average over the thickness total Lagrangian

α-Zr

Standard Rietveld refinement

a/a_0	c/c_0	V/V_0
0.996	0.995	0.988

Pressure (GPa) based on hydrostatic generalized EoS

p_a	p_c	p_V
0.987	1.463	1.138

In plane Rietveld refinement

$E_{0,rr}$	$E_{0,\theta\theta}$	$E_{0,zz}$	$E_{0,v}$
-0.0032	-0.0033	-0.0055	-0.012

Stresses (GPa) based on Hooke's law under pressure

σ_{rr}	$\sigma_{\theta\theta}$	σ_{zz}	p	$\sigma_{rr}-\sigma_{zz}$	$\sigma_y(p)$
-1.07	-1.07	-1.33	1.15	0.26	1.04

CEA

$E_{0,rr}$	$E_{0,\theta\theta}$	$E_{0,zz}$	$E_{0,v}$
-0.00325	-0.00325	-0.01647	-0.023

σ_{rr}	$\sigma_{\theta\theta}$	σ_{zz}	p	$\sigma_{rr}-\sigma_{zz}$	$\sigma_y(p)$
-1.96	-1.96	-3.24	2.39	1.28	1.28

Supplementary Fig. 4: **Comparison of the stresses and elastic strains from the standard Rietveld refinement and CEA for α-Zr.**

strain is

$$\overline{E}_{0,33} = \bar{\tilde{\varepsilon}}_{33} - 0.00664\bar{\tilde{\varepsilon}}_{33}\overline{p} + 0.00013\bar{\tilde{\varepsilon}}_{33}\overline{p^2} + 0.5(0.00013\overline{p^2} - 0.00664\overline{p}) \tag{88}$$

for α-Zr and

$$\overline{E}_{0,33} = \bar{\tilde{\varepsilon}}_{33} - 0.0066\bar{\tilde{\varepsilon}}_{33}\overline{p} + 0.000077\bar{\tilde{\varepsilon}}_{33}\overline{p^2} + 0.5(0.000077\overline{p^2} - 0.0066\overline{p}) \tag{89}$$

for ω-Zr. In obtaining Eqs. (88) and (89), assumptions $\frac{1}{h}\int_0^h \tilde{\varepsilon}_{33}p\,dz \simeq \bar{\tilde{\varepsilon}}_{33}\overline{p}$ and $\frac{1}{h}\int_0^h \tilde{\varepsilon}_{33}p^2\,dz \simeq \bar{\tilde{\varepsilon}}_{33}\overline{p^2}$ are used.

3 The reasons for the difference between stresses and elastic strains from Rietveld refinement and CEA approach

To better illustrate the main sources and reasons for the difference between two approaches, let us calculate averaged over thickness stresses at the sample center, where shear strain, stresses, and m are zero, see Supplementary Fig. 4. Three approaches will be compared.

1. Using standard Rietveld refinement, the ratios of lattice parameters for the chosen sample thickness of α-Zr are $a/a_0 = 0.996$, $c/c_0 = 0.995$, and for the corresponding unit cell volume ratio $V/V_0 = 0.988$. Utilization of the experimental generalized EOSs for a, c, and V results in three different pressures, p_a= 0.987 GPa, p_c=1.463 GPa, and p_V=1.138 GPa, respectively. Difference between these values shows the inconsistency of utilizing EOS ob-

tained under hydrostatic conditions to determine pressure under non-hydrostatic conditions, especially with the axial diffraction.

2. If we use $\bar{E}_{0,rr} = -0.0032$ and $\bar{E}_{0,\theta\theta} = -0.0033$ measured experimentally (which are consistent with $a/a_0 = 0.996$ and c-axis aligned with z-axis), and determine $\bar{E}_{0,zz} = -0.0055$ to have the same $V/V_0 = 0.988$ from the standard Rietveld refinement (since $\bar{E}_{0,zz}$ does not contribute to the X-ray diffraction patterns in the axial geometry), then applying the modified pressure-dependent Hooke's law (assuming $\bar{E}_{0,rr} \approx \bar{E}_{0,\theta\theta} = 0.5(\bar{E}_{0,rr} + \bar{E}_{0,\theta\theta})$), we obtain $\sigma_{rr} = \sigma_{\theta\theta} =$ -1.07 GPa, σ_{zz} =-1.33 GPa, and p=1.15 GPa. Thus, p did not practically change in comparison with p_V. The main problem is that $|\sigma_{zz} - \sigma_{rr}| = 0.26$ GPa, which is much smaller than the yield strength $\sigma_y = 0.82 + 0.19 * 1.15 = 1.04$ GPa at such pressure, which is contradictory.

3. If alternatively, we use the same $\bar{E}_{0,rr} = \bar{E}_{0,\theta\theta} = -0.00325$ and determine $\bar{E}_{0,zz} = -0.01647$ from the developed CEA approach, we obtain $\sigma_{rr} = \sigma_{\theta\theta} = -1.96$ GPa, $\sigma_{zz} = -3.24$ GPa, and $p = 2.39$ GPa. The yield condition $|\sigma_{zz} - \sigma_{rr}| = \sigma_y = 0.82 + 0.19 * 2.38 = 1.28$ GPa is met, i.e., everything is consistent. The main reason for the difference of 1.24 GPa between p=1.15 GPa in the approach #2 and p=2.39 GPa, in which $\bar{E}_{0,zz}$ is determined with CEA to satisfy the yield condition, is that for axial XRD $\bar{E}_{0,zz}$ does not contribute to the XRD patterns, which is neglected in the traditional Rietveld refinement but is taken into account in the developed CEA method. Another conclusion is that standard Rietveld refinement for axial diffraction underestimates volumetric strain, -0.012 instead of -0.023.

Similar results for ω-Zr are presented in Supplementary Fig. 5. While difference in pressure between standard Rietveld refinement and that with CEA is significant (2.2 GPa), the pressure obtained with approach 2 is the same as with the CEA. However, stress components and especially their difference $|\sigma_{rr} - \sigma_{zz}|$ change significantly, by 1.9 GPa.

4 FEM simulations

4.1 Complete system of equations for FEM simulations [8]

Box 1 summarizes all equations derived in [8] in the form used in our simulations. Vectors and tensors are denoted in boldface type, e.g., $\boldsymbol{A} = A_{ij}\boldsymbol{e}_i\boldsymbol{e}_j$, where A_{ij} are components in the Cartesian system with unit basis vectors $\boldsymbol{e}_i$ and summation over the repeated indices is assumed. Expressions $\boldsymbol{e}_i\boldsymbol{e}_j$ and $\boldsymbol{e}_i\boldsymbol{e}_k\boldsymbol{e}_t\boldsymbol{e}_d$ designate the direct or dyadic product of vectors, which represent second- and fourth-rank tensors, respectively. Let $\boldsymbol{A} \cdot \boldsymbol{B} = A_{ik}B_{kj}\boldsymbol{e}_i\boldsymbol{e}_j$ and $\boldsymbol{A} : \boldsymbol{B} = tr(\boldsymbol{A} \cdot \boldsymbol{B}) = A_{ij}B_{ji}$ be the contraction (or scalar product) of tensors over one and two nearest indices, where tr is the trace operation (sum of the diagonal components), and $A_{ik}B_{kj}$ is the matrix product. In the equations, first $\cdot$ is performed, and then :, e. g., $\boldsymbol{A} : \boldsymbol{B} \cdot \boldsymbol{K} = \boldsymbol{A} : (\boldsymbol{B} \cdot \boldsymbol{K})$. The direct (or dyadic) product of two tensors $\boldsymbol{K}$ and $\boldsymbol{M}$ is the tensor $\boldsymbol{KM}$ of rank equal to the sum of the two initial ranks. In particular, for the second-rank tensors $\boldsymbol{K} = K_{ij}\boldsymbol{e}_i\boldsymbol{e}_j$ and $\boldsymbol{M} = M_{kl}\boldsymbol{e}_k\boldsymbol{e}_l$, one has $\boldsymbol{KM} = K_{ij}M_{kl}\boldsymbol{e}_i\boldsymbol{e}_j\boldsymbol{e}_k\boldsymbol{e}_l$. Also, $\boldsymbol{A}_s =: \frac{\boldsymbol{A}+\boldsymbol{A}^t}{2}$ and $\boldsymbol{A}_a =: \frac{\boldsymbol{A}-\boldsymbol{A}^t}{2}$ are respectively the symmetric and anti-symmetric components of $\boldsymbol{A}$, where $'t'$ in the superscript designates the transpose operation defined as $\boldsymbol{A}^t = A_{ji}\boldsymbol{e}_i\boldsymbol{e}_j$, when $\boldsymbol{A} = A_{ij}\boldsymbol{e}_i\boldsymbol{e}_j$.

Box 1. The complete system of equations

ω −Zr

Standard Rietveld refinement

a/a_0	c/c_0	V/V_0
0.958	0.961	0.882

Pressure (GPa) based on hydrostatic generalized EoS

p_a	p_c	p_V
15.2	15.5	15.3

In plane Rietveld refinement

$E_{0,rr}$	$E_{0,\theta\theta}$	$E_{0,zz}$	$E_{0,v}$
-0.0364	-0.0377	-0.0439	-0.118

Stresses (GPa) based on Hooke's law under pressure

σ_{rr}	$\sigma_{\theta\theta}$	σ_{zz}	p	$\sigma_{rr}-\sigma_{zz}$	$\sigma_y(p)$
-17.1	-17.1	-18.3	17.5	1.2	3.1

CEA

$E_{0,rr}$	$E_{0,\theta\theta}$	$E_{0,zz}$	$E_{0,v}$
-0.03705	-0.03705	-0.06102	-0.135

σ_{rr}	$\sigma_{\theta\theta}$	σ_{zz}	p	$\sigma_{rr}-\sigma_{zz}$	$\sigma_y(p)$
-16.5	-16.5	-19.6	17.5	3.1	3.1

Supplementary Fig. 5: **Comparison of the stresses and elastic strains from the standard Rietveld refinement and CEA for ω -Zr.**

Decomposition of the total deformation gradient $\boldsymbol{F}$ into elastic $\boldsymbol{F}_e$ and inelastic $\boldsymbol{F}_i$ contributions:

$$\boldsymbol{F} = \frac{\partial \boldsymbol{r}}{\partial \boldsymbol{r}_0} = \boldsymbol{V}_e \cdot \boldsymbol{R}_e \cdot \boldsymbol{U}_i = \boldsymbol{V}_e \cdot \bar{\boldsymbol{F}}_i; \bar{\boldsymbol{F}}_i = \boldsymbol{R}_e \cdot \boldsymbol{U}_i; \boldsymbol{B}_e = 0.5(\boldsymbol{F}_e \cdot \boldsymbol{F}_e^t - \boldsymbol{I}) = 0.5(\boldsymbol{V}_e^2 - \boldsymbol{I}), \tag{90}$$

where $\boldsymbol{r}$ and $\boldsymbol{r}_0$ are respectively the position vectors of the material point in current and reference configurations. $\boldsymbol{V}_e$ and $\boldsymbol{R}_e$ are from the polar decomposition of $\boldsymbol{F}_e$; $\boldsymbol{U}_i$ is the right stretch tensor from the polar decomposition of $\boldsymbol{F}_i$ and $\boldsymbol{B}_e$ is the elastic Eulerian strain tensor. Decomposition of the deformation rate $\boldsymbol{d}$ into elastic, plastic, and transformation parts:

$$\boldsymbol{d} = \overset{\nabla}{\boldsymbol{B}}_e \cdot \boldsymbol{V}_e^{-2} + 2(\boldsymbol{d} \cdot \boldsymbol{B}_e)_a \cdot \boldsymbol{V}_e^{-2} + \boldsymbol{\gamma} + \bar{\varepsilon}_{t0}\dot{c}\boldsymbol{I} \tag{91}$$

where $\overset{\nabla}{\boldsymbol{B}}_e = \dot{\boldsymbol{B}}_e - 2(\boldsymbol{w} \cdot \boldsymbol{B}_e)_s$ is the Jaumann time derivative of $\boldsymbol{B}_e$, $\boldsymbol{w}$ is the anti-symmetric part of the velocity gradient in the current configuration $\boldsymbol{l}$; $\boldsymbol{\gamma}$ is the plastic part of the deformation rate, $\bar{\varepsilon}_{t0}$ is the volumetric transformation strain, and $\boldsymbol{I}$ is the unit tensor.

The third-order Murnaghan potential:

$$\psi(\boldsymbol{B}_e) = \frac{\lambda + 2G}{2} I_1^2 - 2GI_2 + \left(\frac{l + 2m}{3} I_1^3 - 2mI_1I_2 + nI_3\right), \tag{92}$$

where λ, G, l, m,and n are the Murnaghan material constants of the mixture and I_1, I_2, and I_3 are the invariants of $\boldsymbol{B}_e$ defined as

$$
\begin{aligned}
&I_1 = B_{e11} + B_{e22} + B_{e33};\\
&I_2 = B_{e22}B_{e33} - B_{e23}^2 + B_{e11}B_{e33} - B_{e13}^2 + B_{e22}B_{e11} - B_{e12}^2;\\
&I_3 = \det(B_e).
\end{aligned}
\tag{93}
$$

A simple mixture rule is used to obtain the Murnaghan constants of the mixture

$$
\begin{aligned}
\lambda = (1-c)\lambda_1 + c\lambda_2; \quad G = (1-c)G_1 + cG_2; \quad m = (1-c)m_1 + cm_2;\\
l = (1-c)l_1 + cl_2; \quad n = (1-c)n_1 + cn_2.
\end{aligned}
\tag{94}
$$

Here, the subscripts 1 and 2 designate α- and ω-Zr, c is the concentration of the ω-Zr.

Elasticity rule for the Cauchy (true) stress:

$$
\begin{aligned}
\boldsymbol{\sigma} &= J_e^{-1}(2\boldsymbol{B}_e + \boldsymbol{I}) \cdot \frac{\partial \psi}{\partial \boldsymbol{B}_e}\\
&= J_e^{-1}(2\boldsymbol{B}_e + \boldsymbol{I}) \cdot \left(\lambda I_1 \boldsymbol{I} + 2G\boldsymbol{B}_e + (lI_1^2 - 2mI_2)\boldsymbol{I} + n\frac{\partial I_3}{\partial \boldsymbol{B}_e} + 2mI_1\boldsymbol{B}_e\right),
\end{aligned}
\tag{95}
$$

where $J_e = det\boldsymbol{F}_e$ is the Jacobian determinant of $\boldsymbol{F}_e$. Compact expressions of $\frac{\partial I_1}{\partial \boldsymbol{B}_e}$, $\frac{\partial I_2}{\partial \boldsymbol{B}_e}$, and $\frac{\partial I_3}{\partial \boldsymbol{B}_e}$ are

$$
\frac{\partial I_1}{\partial \boldsymbol{B}_e} = \boldsymbol{I}; \quad \frac{\partial I_2}{\partial \boldsymbol{B}_e} = -\boldsymbol{B}_e + I_1\boldsymbol{I}; \quad \frac{\partial I_3}{\partial \boldsymbol{B}_e} = \boldsymbol{B}_e \cdot \boldsymbol{B}_e - I_1\boldsymbol{B}_e + I_2\boldsymbol{I}. \tag{96}
$$

Yield surface:

$$
\phi = \sqrt{3/2\boldsymbol{s} : \boldsymbol{s}} - (\sigma_{y0} + bp) = 0; \sigma_{y0} = (1-c)\sigma_{y01} + c\sigma_{y02}; b = (1-c)b_1 + cb_2. \tag{97}
$$

Here, $\boldsymbol{s}$ is the deviatoric part of the Cauchy stress $\boldsymbol{\sigma}$, p is the pressure, σ_{y01} and σ_{y02} are the yield strengths in compression of the α- and ω-Zr @$p = 0$, respectively, b_1 and b_2 are their linear pressure hardening coefficients.

Plastic flow rule:

$$
\boldsymbol{\gamma} = |\boldsymbol{\gamma}|\frac{\boldsymbol{s}}{\sqrt{\boldsymbol{s} : \boldsymbol{s}}} = |\boldsymbol{\gamma}|\boldsymbol{n}; |\boldsymbol{\gamma}| = (\boldsymbol{\gamma} : \boldsymbol{\gamma})^{0.5} \text{ when } \phi(\boldsymbol{s}, p, c) = 0 \text{ and } \dot{\phi}(\boldsymbol{s}, p, c) = 0, \tag{98}
$$

i.e., in the elastoplastic region, and $|\boldsymbol{\gamma}|$ is determined from the consistency condition $\dot{\phi}(\boldsymbol{s}, p, c) = 0$; where $|\boldsymbol{\gamma}| = 0$ in the elastic region when $\phi(\boldsymbol{s}, p, c) < 0$ or $\phi(\boldsymbol{s}, p, c) = 0$ and $\dot{\phi}(\boldsymbol{s}, p, c) = 0$.

$$
|\boldsymbol{\gamma}| = \frac{-\left(\frac{\sqrt{1.5}\boldsymbol{s}}{\sqrt{\boldsymbol{s}:\boldsymbol{s}}} + \frac{b}{3}\boldsymbol{I}\right) : \boldsymbol{Y}}{\left(\frac{\sqrt{1.5}\boldsymbol{s}}{\sqrt{\boldsymbol{s}:\boldsymbol{s}}} + \frac{b}{3}\boldsymbol{I}\right) : \boldsymbol{Z} + \frac{\partial \phi}{\partial c}A\sqrt{\frac{2}{3}}} : \boldsymbol{d} \tag{99}
$$

where $\boldsymbol{Y} = \frac{\partial \boldsymbol{\sigma}}{\partial \boldsymbol{B}_e} \cdot \boldsymbol{V}_e^2$, $\boldsymbol{Z} = -\boldsymbol{Y} : \left[\boldsymbol{n} + (\bar{\varepsilon}_{t0}\boldsymbol{I} + \boldsymbol{\gamma}_t)A\sqrt{\frac{2}{3}} + \frac{\partial \boldsymbol{f}}{\partial c}A\sqrt{\frac{2}{3}}\right]$, $\boldsymbol{\sigma} = \boldsymbol{f}(\boldsymbol{B}_e, c)$, and $\frac{dc}{dq} = A(p, q, c)$.

Accumulated plastic strain:

$$\dot{q} = \sqrt{2/3}|\boldsymbol{\gamma}| \tag{100}$$

Stress rate – deformation rate relationship:

$$\overset{\nabla}{\boldsymbol{\sigma}} = \left(\boldsymbol{Y} + \boldsymbol{Z}\frac{-\left(\frac{\sqrt{1.5}\boldsymbol{s}}{\sqrt{\boldsymbol{s}:\boldsymbol{s}}} + \frac{b}{3}\boldsymbol{I}\right) : \boldsymbol{Y}}{\frac{\partial \phi}{\partial \boldsymbol{\sigma}} : \boldsymbol{Z} + \left(\frac{\partial \phi}{\partial c}A + \frac{\partial \phi}{\partial q}\right)\sqrt{\frac{2}{3}}}\right) : \boldsymbol{d} \tag{101}$$

Equilibrium equation:

$$\boldsymbol{\nabla} \cdot \boldsymbol{\sigma} = \boldsymbol{0} \tag{102}$$

4.2 Geometry and boundary conditions

Geometry of DAC is shown in see Fig. 1a in the main text. Axisymmetric problem formulation is considered. Geometry of the sample and the anvil, as well as the boundary conditions, are shown in see Supplementary Fig. 6. They are:

(1) A uniform vertical displacement is applied at the boundary between the top inclined surface of the anvil and Boehler-type seat (line CD). Distributions of stresses or displacements along this surface do not affect fields in the sample and the diamond close to the diamond culet.

(2) At the symmetry axis $r = 0$ (line AB), τ_{rz} and horizontal displacement are zero. At the symmetry plane $z = 0$, shear stress τ_{rz} and vertical displacement are zero.

(3) At the contact surface between the sample and the anvil, an isotropic friction model, described below, is utilized.

(4) Other surfaces not mentioned above are stress-free. Quadrilateral 4-node bilinear axisymmetric finite elements CGAX4R are used in simulations, which are commonly used for large-deformation axisymmetric problems [9]. Our simulations utilize a mesh with 4271 elements.

Evolution of concentration of ω phase $c(r)$ and corresponding volumetric transformation strain $\bar{\varepsilon}_{t0}\dot{c}\boldsymbol{I}$ ($\bar{\varepsilon}_{t0} = -0.0158$) are introduced homogeneously along the z-coordinate in our FEM problem formulation for each loading step.

Friction model:

At the culet portion of the diamond $r \leq r_c$, the contact shear stress is given by

$$\tau_c = m'\tau_y(p) \qquad \text{for } r \leq r_c \tag{103}$$

The distribution of $m'(r)$ is obtained from the analytical solution (Supplementary Fig. 7).

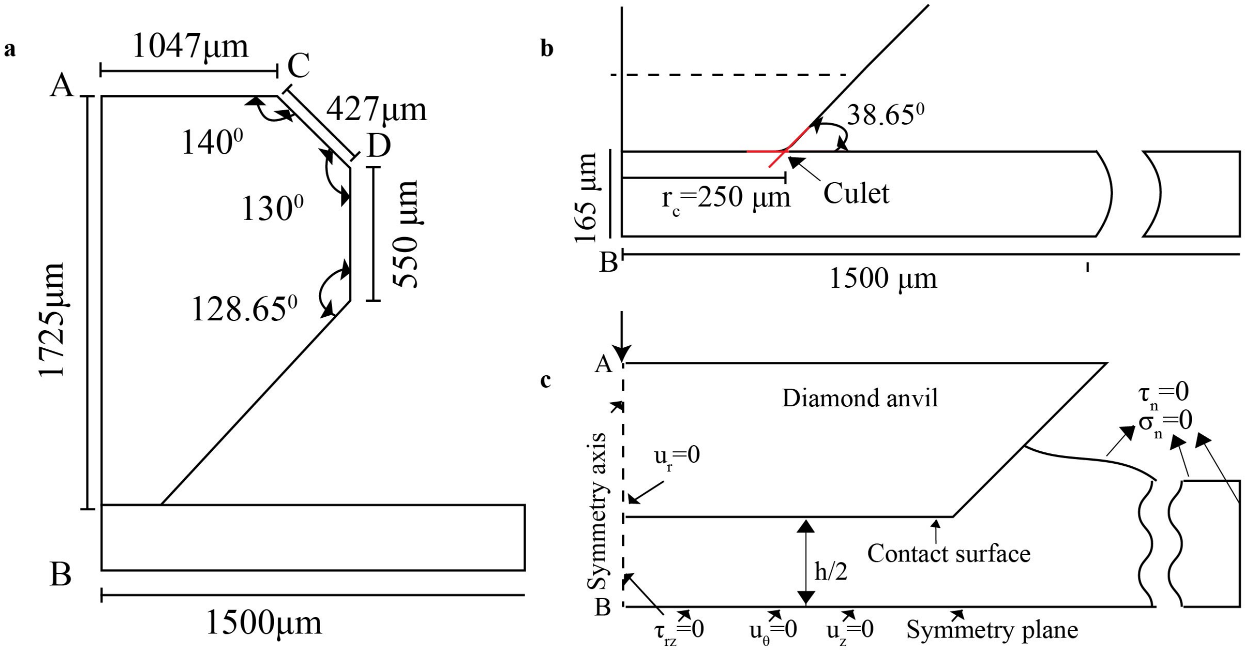

Supplementary Fig. 6: **Details of the geometrical model of the DAC experiment used in FEM: a** Description of the one half of diamond dimensions in the natural configuration. **b** Geometric parameters of one half of sample and the angle between culet and non-culet diamond surfaces. **c** Boundary conditions and the schematic of sample in the deformed configuration.

At the inclined portion of the sample-diamond contact surface, the critical shear is governed by the combined Coulomb friction $\tau_{cr} = \mu(\sigma_c)\sigma_c$ and Eq. (103), where σ_c is the contact normal stress. There is complete cohesion between the sample and the anvil unless shear stress τ_c reaches the critical value:

$$\tau_c < \tau_{cr} = min\left[\mu(\sigma_c)\sigma_c, m'(r_c)\tau_y(p)\right] \rightarrow cohesion, \quad (104)$$

where $m^{'} = m^{'}(r_c)$ is determined at $r = r_c$. When friction stress reaches τ_{cr},

$$\tau_c = \tau_{cr} = min[\mu(\sigma_c)\sigma_c, m'(r_c)\tau_y(p)] \rightarrow sliding, \quad (105)$$

contact sliding occurs.

4.3 Nonlinear elastic equations and material properties for single-crystal diamond anvil

The constitutive response of diamond is modeled using fourth-order nonlinear anisotropic elastic potential energy given by Vekilov et al. [10]:

$$\begin{aligned}\psi =&\frac{1}{2}c_{11}(\eta_1^2+\eta_2^2+\eta_3^2)+c_{12}(\eta_1\eta_2+\eta_2\eta_3+\eta_1\eta_3)+\frac{1}{2}c_{44}(\eta_4^2+\eta_5^2+\eta_6^2)+\frac{1}{6}c_{111}(\eta_1^3+\eta_2^3+\\&\eta_3^3)+\frac{1}{2}c_{112}[\eta_1^2(\eta_2+\eta_3)+\eta_2^2(\eta_1+\eta_3)+\eta_3^2(\eta_1+\eta_2)]+c_{123}\eta_1\eta_2\eta_3+c_{456}\eta_4\eta_5\eta_6+\\&\frac{1}{2}c_{144}(\eta_1\eta_4^2+\eta_2\eta_5^2+\eta_3\eta_6^2)+\frac{1}{2}c_{155}[\eta_4^2(\eta_2+\eta_3)+\eta_5^2(\eta_1+\eta_3)+\eta_6^2(\eta_1+\eta_2)]+\\&\frac{1}{24}c_{1111}(\eta_1^4+\eta_2^4+\eta_3^4)+\frac{1}{6}c_{1112}[\eta_1^3(\eta_2+\eta_3)+\eta_2^3(\eta_1+\eta_3)+\eta_3^3(\eta_1+\eta_2)]+\frac{1}{4}c_{1144}(\eta_1^2\eta_4^2+\\&\eta_2^2\eta_5^2+\eta_3^2\eta_6^2)+\frac{1}{4}c_{1122}(\eta_1^2\eta_2^2+\eta_2^2\eta_3^2+\eta_3^2\eta_1^2)+\frac{1}{2}c_{1123}\eta_1\eta_2\eta_3(\eta_1+\eta_2+\eta_3)+\\&\frac{1}{4}c_{1155}[\eta_1^2(\eta_6^2+\eta_5^2)+\eta_2^2(\eta_6^2+\eta_4^2)+\eta_3^2(\eta_5^2+\eta_4^2)]+\frac{1}{2}c_{1255}[\eta_1\eta_2(\eta_4^2+\eta_5^2)+\eta_3\eta_2(\eta_6^2+\\&\eta_5^2)+\eta_1\eta_3(\eta_6^2+\eta_4^2)]+\frac{1}{2}c_{1266}(\eta_1\eta_2\eta_6^2+\eta_2\eta_3\eta_4^2+\eta_1\eta_3\eta_5^2)+c_{1456}\eta_4\eta_5\eta_6(\eta_1+\eta_2+\eta_3)+\\&\frac{1}{24}c_{4444}(\eta_4^4+\eta_5^4+\eta_6^4)+\frac{1}{4}c_{4455}(\eta_4^2\eta_5^2+\eta_6^2\eta_5^2+\eta_4^2\eta_6^2),\end{aligned} \tag{106}$$

where $\eta_1=E_{e11}$, $\eta_2=E_{e22}$, $\eta_3=E_{e33}$, $\eta_4=2E_{e23}$, $\eta_5=2E_{e31}$,and $\eta_6=2E_{e12}$ are the Lagrangian strains. Based on the elasticity law, the Cauchy stress in the diamond can be obtained using:

$$\boldsymbol{\sigma}=\frac{1}{J}\boldsymbol{F}_e\cdot\frac{\partial\psi}{\partial\boldsymbol{E}_e}\cdot\boldsymbol{F}_e^t. \tag{107}$$

Here J is the Jacobian determinant of $\boldsymbol{F}_e$. All the elastic constants of diamond are taken from Telichko et al. [11] and they are as follows (all in GPa):

$$\begin{aligned}&c_{11}=1081.9, c_{12}=125.2, c_{44}=578.6;\\&c_{111}=-7611, c_{112}=-1637, c_{123}=604, c_{144}=-199, c_{166}=-2799, c_{155}=-2799,\\&c_{456}=-1148, c_{1111}=26687, c_{1112}=9459, c_{1122}=6074, c_{1123}=-425, c_{1144}=-1385,\\&c_{1155}=10741, c_{1255}=-264, c_{1266}=8192, c_{1456}=487, c_{4444}=11328, c_{4455}=528.\end{aligned} \tag{108}$$

4.4 Elastic properties of polycrystalline α- and ω-Zr

The elastic constitutive response of polycrystalline Zr is modeled using the third-order nonlinear Murnaghan potential Eq. (92). Out of 5 elastic constants in the Murnaghan potential, 2 elastic constants, Lame constant λ and shear modulus G, are related to the quadratic in $\boldsymbol{B}_e$ terms, and the rest, l, m and n, are related to the cubic in $\boldsymbol{B}_e$ terms. These constants are calibrated using the bulk modulus K and its pressure derivative $\frac{dK}{dp}@p=0$ obtained from the pressure-volume relationships in hydrostatic DAC experiments, and the shear modulus G and its pressure-derivative $\frac{dG}{dp}@p=0$ are taken from the experimental results [12,13]. The

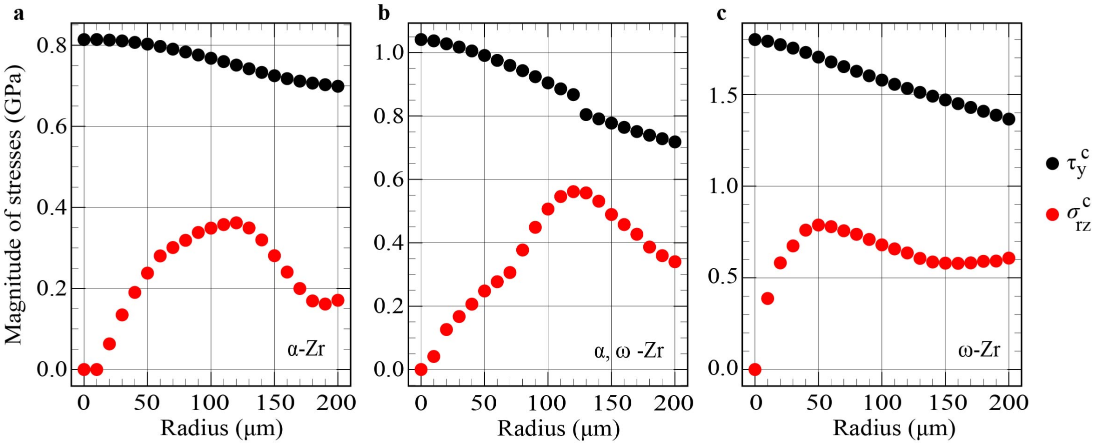

Supplementary Fig. 7: **Radial distributions of shear stress and yield strength in shear a** for pure α-Zr, **b** for the mixture of α- and ω-Zr and **c** for the pure ω-Zr obtained using an analytical model and experimentally measured radial $\bar{E}_{0,rr}$ and azimuthal $\bar{E}_{0,\theta\theta}$ strain distributions.

expressions relating the Murnaghan constants λ, G, l, m, n and K, G, $\frac{dK}{dp}$ and $\frac{dG}{dp}$ @ $p = 0$ are:

$$K = \frac{3\lambda + 2G}{3}; \quad \frac{dK}{dp} = K' = -\frac{2(9l + n)}{9K}; \quad \frac{dG}{dp} = G' = \frac{-2G - 6K - 6m + n}{6K}. \tag{109}$$

It can be seen there are only 2 equations to solve for the 3 third-order constants. Therefore, there is an indeterminacy of degree 1. However, it can be easily shown for any pressure, that when the deviatoric part of the superposed deformation is small, the stresses and energy can be written in terms of just K, G, K', and G'. Therefore, one of the constants, l, m, or n, can be chosen arbitrarily, and the other two are determined from Eq. (109). The constants that are used are (all in GPa):

$$\begin{aligned} \lambda = 68.11, \quad G = 36.13, \quad l = -147.01, m = -122.75, \quad n = -100 \qquad & \text{for } \alpha - Zr; \\ \lambda = 72.33, \quad G = 45.1, \quad l = -149.56, m = -179.53, \quad n = -4 \qquad & \text{for } \omega - Zr. \end{aligned} \tag{110}$$

5 Friction stress and rules for α and ωZr and their mixture

Friction along the sample-diamond contact surface in the DAC experiment plays a central role in generating high pressure in the sample. However, the magnitude of the friction force along the contact surface cannot be measured. Traditionally [5, 14, 15], the yield strength in shear was assumed to be equal to the sliding friction force. But it was reported recently in [16,17] that the sliding friction force is significantly lower than the yield strength in shear. This important deduction, coupled with the elusive nature of the mechanism of the sliding friction at high pressure, posed an important problem to find boundary conditions for FEM solution and increased the degree of indeterminacy of all fields in a sample. The problem is resolved by using the results of analytical model that gives the distributions of contact shear stress (Supplementary Fig. 7). For the mixture of α+ω Zr, there is a jump in yield strength in shear of mixture, which is due to the jump in the concentration of the ω-Zr phase (See Fig. 2 in main text).

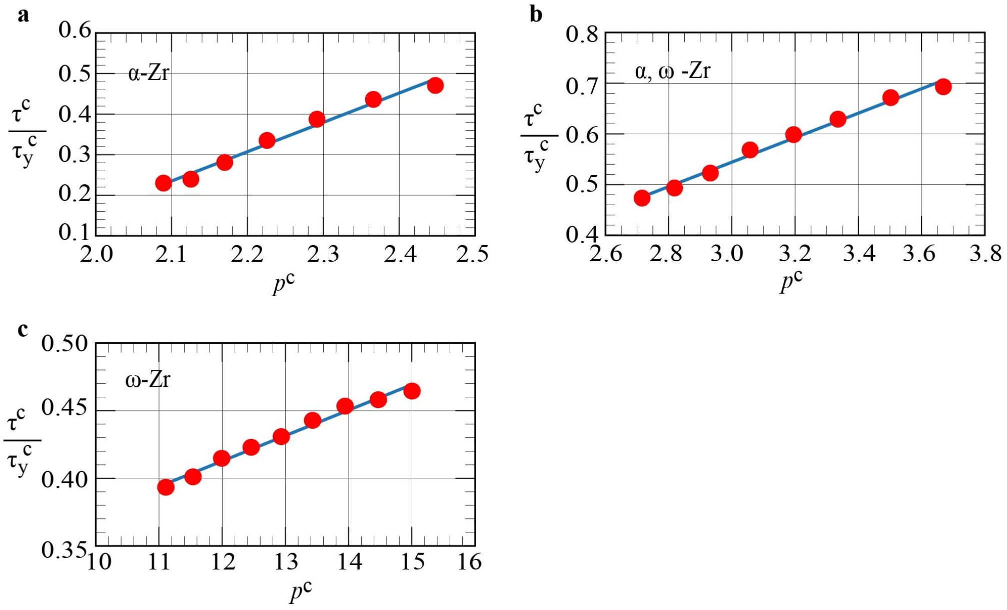

Supplementary Fig. 8: **Pressure dependence of the contact friction stress τ_c normalized by the yield strength in shear τ_y^c at the same point.** Results are presented for **a** pure α-Zr at $p_{max} = 3.09$ GPa, **b** mixture of α- and ω-Zr at $p_{max} = 4.77$ GPa, and **c** pure ω-Zr at $p_{max} = 17.55$ GPa.

To find friction sliding laws, we must exclude regions near the symmetry axis. At the symmetry axis, friction stress is zero due to symmetry, which leads to zero or very small sliding near the symmetry. We also exclude region near the culet edge, where character of the plastic flow significantly changes.

The best linear fits for the contact friction stress τ_c normalized by the yield strength in shear τ_y^c at the same point for pure-ω Zr in the region r =60 μm to 140 μm, for the mixture of α and ω Zr in the region r=130 μm to 200 μm, and for the pure-α Zr in the region r=130 μm to 190 μm are (Supplementary Fig. 8):

$$\begin{aligned}
&\left(\frac{\tau_c}{\tau_y^c}\right)_{\omega} = 0.186 + 0.018 p^c \quad \text{for } 11.1 \leq p^c(GPa) \leq 15.0; \quad 60 \leq r(\mu m) \leq 140; \\
&\left(\frac{\tau_c}{\tau_y^c}\right)_{\alpha+\omega} = 0.179 + 0.241 p^c \quad \text{for } 2.7 \leq p^c(GPa) \leq 3.7; \quad 130 \leq r(\mu m) \leq 200; \\
&\left(\frac{\tau_c}{\tau_y^c}\right)_{\alpha} = -1.282 + 0.722 p^c \quad \text{for } 2.0 \leq p^c(GPa) \leq 2.45; \quad 130 \leq r(\mu m) \leq 190;
\end{aligned} \tag{111}$$

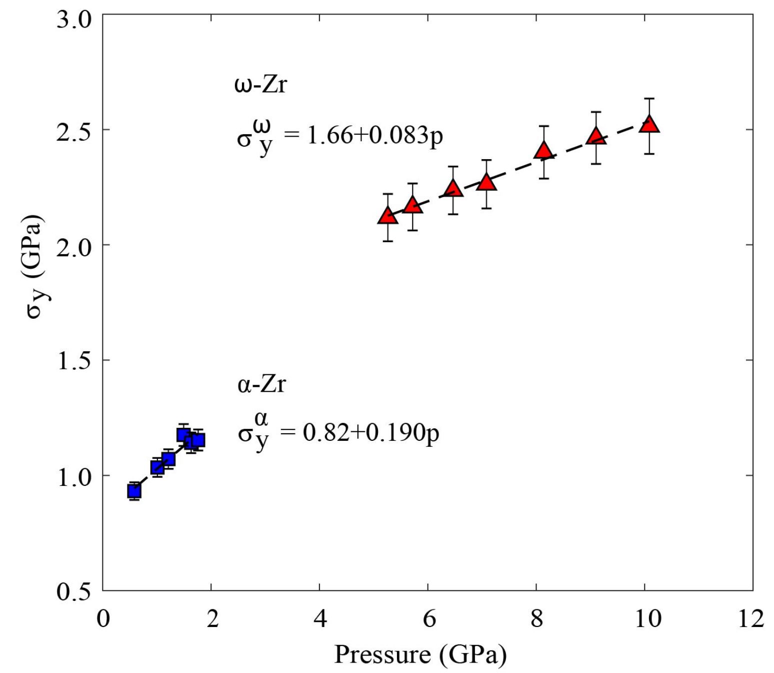

Supplementary Fig. 9: **Pressure dependence of yield strength in compression of α- and ω-Zr.**

6 Pressure-dependence of the yield strengths of α and ωZr

The yield strengths of α and ω phases of Zr were estimated using the peak broadening method [1] near the center of a sample (see Supplementary Fig. 9):

$$\sigma_y^\alpha = 0.82 + 0.19p \text{ (GPa)} \quad \text{and} \quad \sigma_y^\omega = 1.66 + 0.083p \text{ (GPa)}$$

It is worth mentioning here that Zhao et al. [1] reported the yield strengths of α and ω phases as 0.18 GPa and 1.18 GPa at ambient pressure, respectively. The reason that our values of the yield strengths are significantly higher is that our Zr sample was subjected to large preliminary plastic deformation until saturation of the strain hardening, while Zhao et al. performed experiments on an annealed Zr sample.

7 Equation of state under hydrostatic loading

Experiments under hydrostatic loading were performed for comparison and to determine the equation of state for both phases. The $\alpha \rightarrow \omega$ PT started at pressure 5.4 GPa and finished at 6.6 GPa. The 3rd order Birch-Murnaghan equation of state (EOS) fitting of pressure-volume data for α and ω phases of Zr provides: initial (ambient) volumes $V_0 = 23.269\,\text{Å}^3$ and $34.306\,\text{Å}^3$ (per formula unit); bulk moduli $K_0 = 93.55$ GPa and 102.4 GPa @ $p = 0$, and pressure derivative of bulk moduli K'=3.0 and 2.93 @ $p = 0$, respectively. ω-Zr is retained at ambient pressure on complete pressure release. Components of the deformation gradient $\boldsymbol{F}_*(p)$ determined in the hydrostatic experiments for α and ω phases are presented in Eqs. (4)- (7).

Supplementary figures

Supplementary Fig. 10: Schematic illustration of estimation of elastic strains in radial $E_{0,rr}$ and azimuthal directions $E_{0,\theta\theta}$.
Supplementary Fig. 11: 2D stress contours for 3 loading cases.
Supplementary Fig. 12: Schematics of DAC assemblies for which the developed approach is applicable.

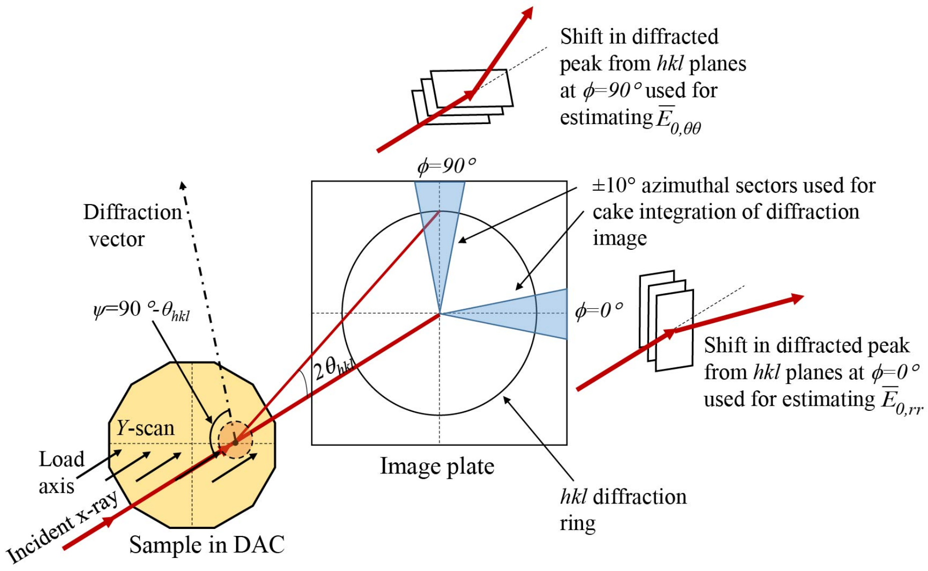

Supplementary Fig. 10: **Schematic illustration of estimation of elastic strains in radial $\bar{E}_{0,rr}$ and azimuthal $\bar{E}_{0,\theta\theta}$ directions at each scanning position.**

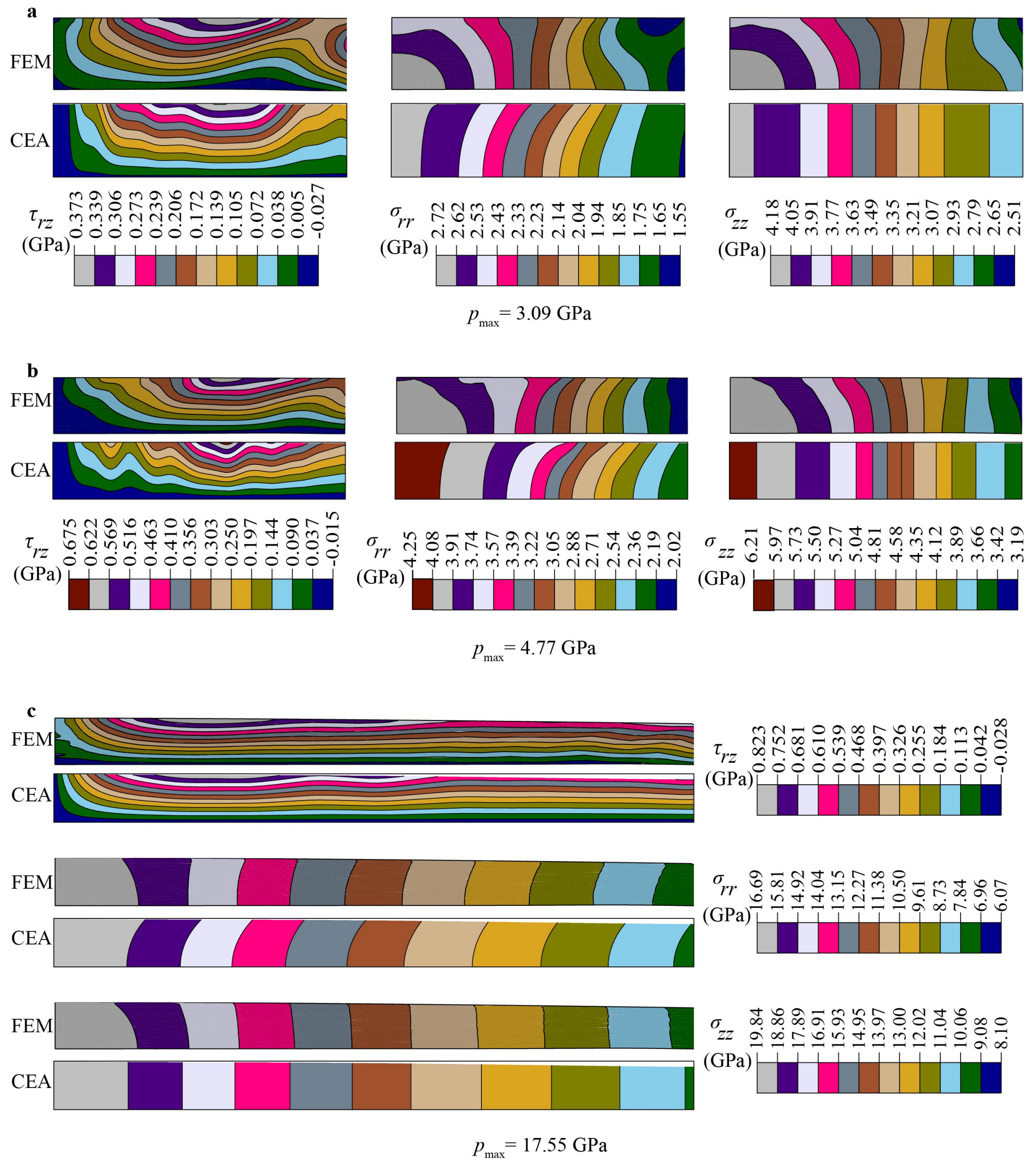

Supplementary Fig. 11: **2D stress contours for three loading cases from FEM and analytical solutions.** **a** Results for almost pure $\alpha - Zr$ at $p_{max} = 3.09$ GPa. **b** Results for mixture of $\alpha-$ and ω- Zr at $p_{max} = 4.77$ GPa. **c** Results for pure $\omega - Zr$ at $p_{max} = 17.55$ GPa.

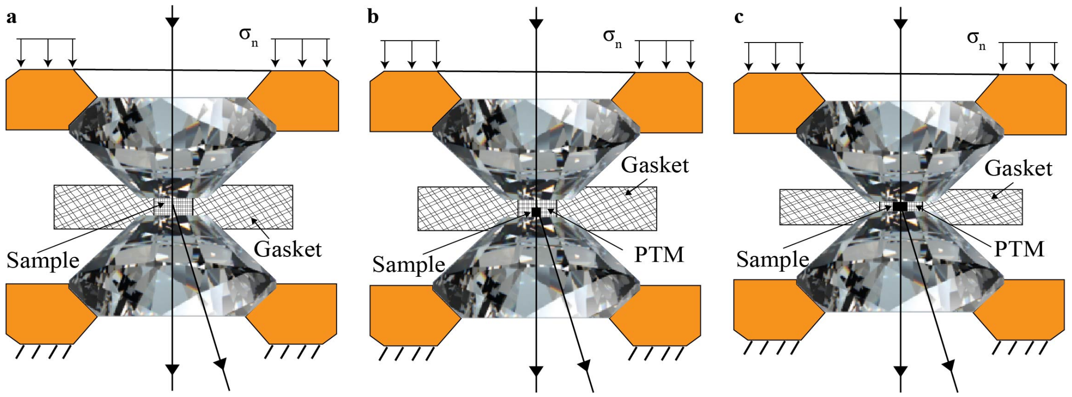

Supplementary Fig. 12: **Schematics of DAC assemblies for which the developed approach is applicable.** **a** Solid sample within a gasket without a hydrostatic medium (e.g., like in [18, 19] with FEM simulations in [20] or any powder material). **b** Solid sample within a gasket with hydrostatic pressure-transmitting medium (PMT) after their solidification (e.g., like in [21–24]). **c** The same as in b but after the sample is directly compressed by anvils, from the beginning or above some load (e.g., like in [18,21,25,26]). All fields in the solidified pressure-transmitting medium and gasket can be studied in the same experiment. The developed approach can be extended for rotational DAC when torque is applied in (a)-(c).

Supplementary References

[1] Zhao, Y. & J. Zhang, Enhancement of yield strength in zirconium metal through high-pressure induced structural phase transition. *Applied Physics Letters*, **91**, 201907 (2007).

[2] Levitas, V.I. Nonlinear elasticity of prestressed single crystals at high pressure and various elastic moduli. *Physical Review B* **104**, 214105 (2021).

[3] Fisher, E. S., Manghnani, M. H., & Sokolowski, T. J. Hydrostatic Pressure Derivatives of the Single-Crystal Elastic Moduli of Zirconium. *J. Appl. Phys.* **41**, 2991 (1970).

[4] Burakovsky, L. private communication (2021).

[5] Levitas, V. I. Large Deformation of Materials with Complex Rheological Properties at Normal and High Pressure (Nova Science Publishers, New York, 1996).

[6] Lin F., Levitas V.I., Pandey K.K., Yesudhas S., & Park C. Laws of high-pressure phase and nanostructure evolution and severe plastic plastic flow. DOI: https://doi.org/10.21203/rs.3.rs-1998605/v1 (2022).

[7] Hill, R.. The Mathematical Theory of Plasticity (Oxford University Press, Oxford, 1998).

[8] Feng, B. & Levitas, V. I. Coupled elastoplasticity and plastic strain-induced phase transformation under high pressure and large strains: Formulation and application to BN sample compressed in a diamond anvil cell. *Int. J. Plast.* **96**, 156-181 (2017).

[9] Dunne, F. & Petrinic, N. Introduction to computational plasticity (OUP Oxford, 2005).

[10] Vekilov, Y. K., Krasilnikov, O. M. & Lugovskoy, A. V. Elastic properties of solids at high pressure. *Physics-Uspekhi*, **58**, 1106 (2015).

[11] Telichko, A. V., Erohin, S. V., Kvashnin, G. M., Sorokin, P. B., Sorokin, B. P. & Blank, V. D. Diamond's third-order elastic constants: Ab initio calculations and experimental investigation. *Journal of materials science*, **52**, 3447-3456 (2017).

[12] Liu, W., Li, B., Wang, L., Zhang, J. & Zhao, Y. Elasticity of ω-phase zirconium. *Physical Review B*, **76**, 144107 (2007).

[13] Liu, W., Li, B., Wang, L., Zhang, J. & Zhao, Y. Simultaneous ultrasonic and synchrotron x-ray studies on pressure induced α-ω phase transition in zirconium. *Journal of Applied Physics*, **104**, 076102 (2008).

[14] Jeanloz, R., Godwal, B. K. & Meade, C. Static strength and equation of state of rhenium at ultra-high pressures. *Nature* **349**, 687-689 (1991).

[15] Meade, C. & Jeanloz, R. The strength of mantle silicates at high-pressures and room-temperature - implications for the viscosity of the mantle. *Nature* **348**, 533-535 (1990).

[16] Levitas, V. I., Kamrani, M. & Feng, B. Tensorial stress-strain fields and large elastoplasticity as well as friction in diamond anvil cell up to 400 GPa. *NPJ Computational Materials* **5**, 94 (2019).

[17] Pandey, K. K. & Levitas, V. I. In situ quantitative study of plastic strain-induced phase transformations under high pressure: Example for ultra-pure Zr. *Acta Materialia*, **196**, 338-346 (2020).

[18] Jenei, Z., O'Bannon, E. F., Weir, S. T., Cynn, H., Lipp, M. J. & Evans, W. J. Single crystal toroidal diamond anvils for high pressure experiments beyond 5 megabar. *Nature communications* **9**, 3563 (2018).

[19] Ji, C., Levitas, V. I., Zhu, H., Chaudhuri, J., Marathe, A. & Ma, Y. Shear-induced phase transition of nanocrystalline hexagonal Boron Nitride to Wurtzitic structure at room temperature and low pressure. *Proceedings of the National Academy of Sciences of the United States of America* **109**, 19108-19112 (2012).

[20] Feng, B. & Levitas, V. I. Pressure self-focusing effect and novel methods for increasing the maximum pressure in traditional and rotational diamond anvil cells. *Sci. Rep.* **7**, 45461 (2017).

[21] Dubrovinsky, L., Dubrovinskaia, N., Prakapenka, V. B. & Abakumov, A. M. Implementation of micro-ball nanodiamond anvils for high-pressure studies above 6 Mbar. *Nature Communications* **3**, 1163 (2012).

[22] Dias, R. P. & Silvera, I. F. Observation of the Wigner-Huntington transition to metallic Hydrogen. *Science* **355**, 715-718 (2017).

[23] Barge, N. V. & Boehler, R. Effect of non-hydrostaticity on the α-ε transition of Iron. *High Pressure Research* **6**, 133–140 (2006).

[24] Hsieh, S., Bhattacharyya, P., Zu, C., Mittiga, T., Smart, T. J., Machado, F., Kobrin, B., Höhn, T. O., Rui, N. Z., Kamrani, M., Chatterjee, S., Choi, S., Zaletel, M., Struzhkin, V. V., Moore, J. E., Levitas, V. I., Jeanloz, R. & Yao, N. Y. Imaging stress and magnetism at high pressures using a nanoscale quantum sensor. *Science* **366**, 1349-1354 (2019).

[25] Dubrovinsky, L., Khandarkhaeva, S., Fedotenko, T., Laniel, D., Bykov, M., Giacobbe, C., Lawrence Bright, E., Sedmak, P., Chariton, S., Prakapenka, V., Ponomareva, A. V., Smirnova, E. A., Belov, M. P., Tasnadi, F., Shulumba, N., Trybel, F., Abrikosov, I. A. & Dubrovinskaia, N. Materials synthesis at terapascal static pressures. *Nature* **605**, 274–278 (2022).

[26] Ding, Y., Sun, Y., Jiang, S., Huang, X. & Cui, T. Frontier in the diamond anvil cell techniques for ultrahigh pressure generation. *Journal of Physics: Condensed Matter* **35**, 313002 (2023).
